# Time Series Analysis of the *Bacillus subtilis* Sporulation Network Reveals Low Dimensional Chaotic Dynamics

**DOI:** 10.3389/fmicb.2016.01760

**Published:** 2016-11-07

**Authors:** Paola Lecca, Ivan Mura, Angela Re, Gary C. Barker, Adaoha E. C. Ihekwaba

**Affiliations:** ^1^Department of Mathematics, University of TrentoTrento, Italy; ^2^Department of Industrial Engineering, Universidad de los AndesBogotá, Colombia; ^3^Laboratory of Translational Genomics, Centre for Integrative Biology, University of TrentoTrento, Italy; ^4^Gut Health and Food Safety, Institute of Food ResearchNorwich, UK

**Keywords:** systems biology, computational modeling, sensitivity analysis, low dimensional chaos, signal transduction, sporulation, *Bacillus subtilis*

## Abstract

Chaotic behavior refers to a behavior which, albeit irregular, is generated by an underlying deterministic process. Therefore, a chaotic behavior is potentially controllable. This possibility becomes practically amenable especially when chaos is shown to be low-dimensional, i.e., to be attributable to a small fraction of the total systems components. In this case, indeed, including the major drivers of chaos in a system into the modeling approach allows us to improve predictability of the systems dynamics. Here, we analyzed the numerical simulations of an accurate ordinary differential equation model of the gene network regulating sporulation initiation in *Bacillus subtilis* to explore whether the non-linearity underlying time series data is due to low-dimensional chaos. Low-dimensional chaos is expectedly common in systems with few degrees of freedom, but rare in systems with many degrees of freedom such as the *B. subtilis* sporulation network. The estimation of a number of indices, which reflect the chaotic nature of a system, indicates that the dynamics of this network is affected by deterministic chaos. The neat separation between the indices obtained from the time series simulated from the model and those obtained from time series generated by Gaussian white and colored noise confirmed that the *B. subtilis* sporulation network dynamics is affected by low dimensional chaos rather than by noise. Furthermore, our analysis identifies the principal driver of the networks chaotic dynamics to be sporulation initiation phosphotransferase B (Spo0B). We then analyzed the parameters and the phase space of the system to characterize the instability points of the network dynamics, and, in turn, to identify the ranges of values of Spo0B and of the other drivers of the chaotic dynamics, for which the whole system is highly sensitive to minimal perturbation. In summary, we described an unappreciated source of complexity in the *B. subtilis* sporulation network by gathering evidence for the chaotic behavior of the system, and by suggesting candidate molecules driving chaos in the system. The results of our chaos analysis can increase our understanding of the intricacies of the regulatory network under analysis, and suggest experimental work to refine our behavior of the mechanisms underlying *B. subtilis* sporulation initiation control.

## 1. Introduction

Bacterial spores are important contaminants in food, and the spore forming bacteria are often implicated in food safety and food quality considerations (Carlin, 2011). Most microbial spore forming bacteria respond to stress (e.g., nutrient deprivation) by inducing the expression of an appropriate suit of adaptive (stress-response) genes to help them cope with adverse environmental circumstances; an extreme example is endospore formation (Ihekwaba et al., 2014).

Since sporulation is an energy consuming process that requires a significant reorganization of cellular activity, the decision to commit to spore formation is subject to the result of integration of multiple signals by a complex gene regulation network.

The initiation of sporulation is one of the decisive moments in spore formation, as exemplified by the bacterium *Bacillus subtilis*. The changes in gene expression and morphology induced by sporulation are regulated in *B. subtilis* by a complex network involving more than 120 genes (Stragier and Losick, 1996; Fawcett et al., 2000).

The DNA-binding protein Spo0A is the master regulator for entry into sporulation in *B. subtilis*. The concentration level and the phosphorylation state determine the ability of Spo0A to alter transcription. Upon phosphorylation, Spo0A undergoes an allosteric change that re-orientates a phenylalanine residue and allows the molecule to bind DNA (Muchová et al., 2004) and activate key genes that drive the positive regulation of sporulation, particularly the *spoIIA*, *spoIIE*, and *spoIIG* genes involved in establishing compartment-specific transcription under the control of σF (*spoIIA* operon and the *spoIIE* gene) and σE (*spoIIG* operon) (Satola et al., 1991, 1992; York et al., 1992). Phosphorylated Spo0A also acts as a repressor, blocking the expression of the *abrB* gene. This repression has the consequence of setting up a self-reinforcing cycle that contributes to the further accumulation of Spo0A at the start of sporulation (Fujita and Losick, 2005; Tojo et al., 2013). Indeed, the inhibition that phosphorylated Spo0A exerts on *abrB* gene expression leads to the depletion of the AbrB protein from the cell and to the accumulation of σH, with the net result of enhancing the expression of KinA, Spo0F and of the Spo0A gene itself (Strauch et al., 1993; Tojo et al., 2013).

Spo0A activation is under the control of a complex network capable of integrating diverse physiological and environmental signals, and relaying signals through a three-level phosphorelay down to the response regulator Spo0A. Various mathematical models of *B. subtilis* sporulation mechanisms can be found in the literature, among the most recent ones we mention (Kuchina et al., 2011; Sen et al., 2011; Narula et al., 2012; Kothamachu et al., 2013; Vishnoi et al., 2013; Ihekwaba et al., 2014).

This paper is based on the model proposed in Ihekwaba et al. (2014), which integrates most of previous mathematical modeling works on *B. subtilis* sporulation initiation. The model we consider encodes the relationships among the time-dependent concentrations of sporulation signals, histidine kinases, phosphorelay proteins and sporulation initiation proteins in the form of a deterministic differential model having 27 variables. Simulation of the differential equations via numerical integration provides predictions about the evolution of the *B. subtilis* sporulation initiation regulation network, given the initial state of variables. Ihekwaba et al. (2014) also performed a sensitivity analysis of the model to explore the set of possible behaviors with varying the values of its parameters (i.e., the kinetic rate constants).

In this paper, we continue the analysis of model behavior, with the aim of investigating whether the time series of the variables, as predicted by the differential equations model, are affected by deterministic chaos, or simply *chaos*. A chaotic system is a system that is predictable up until a given time, after which it becomes unpredictable (i.e., long term unpredictability) due to its sensitivity to initial conditions (Kellert, 1993). Even if the initial state is known at a very accurate level of detail, any imprecision in its quantification, no matter how small, grows quickly (exponentially) with time, rendering long-term prediction impossible.

Identifying chaos and its drivers in a biological system provides useful information (i) to understand the origins of the observed dynamics (Weiss et al., 1994; Lecca et al., 2016), and (ii) to shed light into the control mechanisms that a biological system may have implemented to maintain a stable activity even when subject to perturbations of its initial conditions (Sinha, 1997). Both chaotic dynamics and stochastic dynamics exhibit a complex phase space structure and are not predictable, but chaos is not stochastic noise (Lecca et al., 2016). Indeed, a chaotic dynamics is governed by deterministic laws in which no randomness is involved, whereas a stochastic dynamics is governed by rules involving random variables. As a consequence, if the laws and the drivers of the dynamics of a chaotic system are known, its unpredictability can potentially be controlled (Sinha, 1997; Lai, 2014).

*Low-dimensional* chaos occurs when a reduced number of contributing species are responsible for the complex dynamics. Such a low-dimensional chaos is of particular interest in biology (Skinner, 1994; Kaneko, 2006; Vasseur, 2015). Since in a biological system affected by low-dimensional chaos the variables governing the spatial and temporal dynamics are few in number, a low-energy control of unpredictability of the system dynamics can be implemented and a simpler model of a complex dynamics can be provided. Low-dimensional chaos is expected to be common in systems with few degrees of freedom (Skinner, 1994), but is expected to be rare in systems with many degree of freedom such as the sporulation network of *B. subtilis*. The results of our analysis show that only few molecular species are contributing to the appearance of deterministic chaos in the dynamics of the modeled network.

## 2. The model

In Ihekwaba et al. (2014), a mathematical model of the network regulating *Bacillus subtilis* sporulation initiation was proposed. The model represents at the molecular level the sequence of events that lead to the activation of the early genes under control of the master regulator molecule Spo0A, distilling and extending the results obtained in various modeling studies focused on systems where sporulation is induced by an artificial inductor, the Isopropyl-D-1-thiogalactopyranoside (IPTG) (see for instance Narula et al., 2012), and modeling as well the induction of sporulation that occurs in wild-type cells.

**Figure 1:**
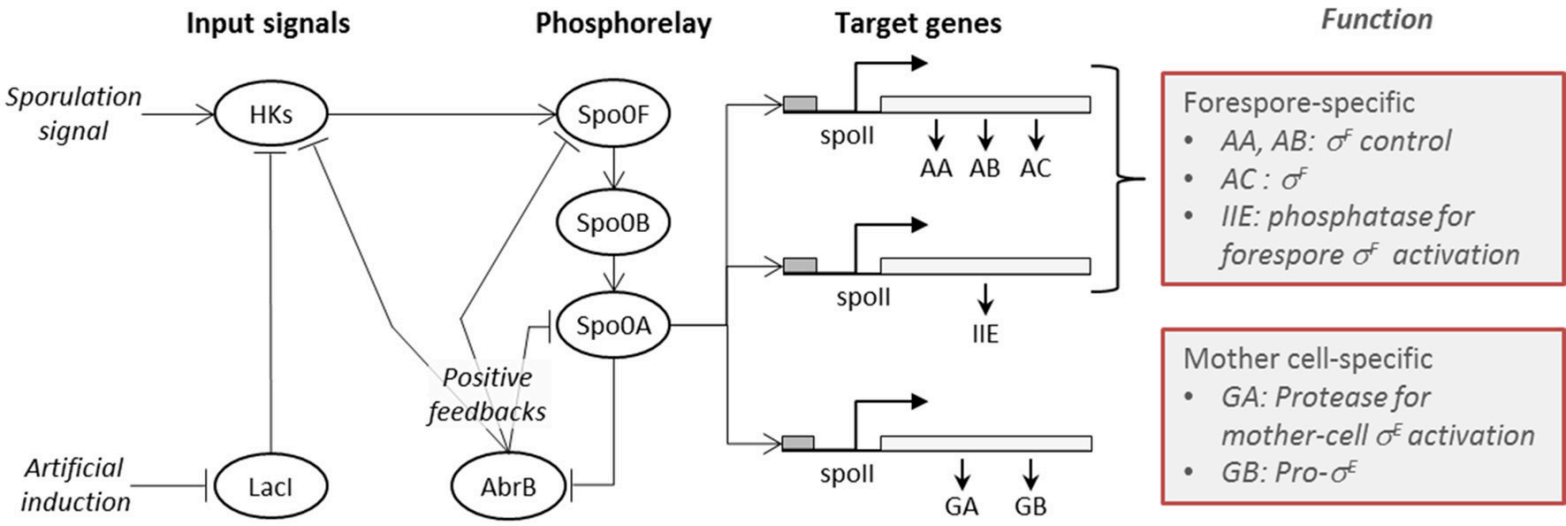
**The sporulation initiation network in *B. subtilis* is activated by signals that first cause the activation of histidine-kinases (HKs)**. This can occur either via the direct accumulation of the natural sporulation signals in the cell or via artificial induction (IPTG), both which have been considered in our modeling. The activated HKs transfer phosphor groups to the phosphorelay mediator proteins Spo0F and Spo0B, until activation of the transcription factor Spo0A. The phosphorylated form of the master regulator protein Spo0A activates the genes (spolla, spolle, and spollg) that govern forespore and mother cell specific transcription factors and exerts a positive feedback on phosphorelay components through the repression of *abrB* gene expression.

A high-level diagrammatic description of the molecular network governing sporulation initiation in *B. subtilis* is provided in Figure 1, where pointed arrows represent activation and blunt arrows indicate repression.

The *B. subtilis* sporulation network model considered in this study is the published model by Lecca et al. (2016) and Ihekwaba et al. (2014). It follows the topology of the network shown in Figure 1, thereby encompassing three distinct sub-models:

1. input signal, representing the sporulation initiation processes induced by the signals on the histidine kinases;
2. phosphorelay, encoding the signal transduction along the phosphorelay components, from histidine kinases downwards to the master regulator Spo0A;
3. gene expression, modeling the target gene expression activation operated by the activated effector Spo0A.

In the following section, we explain the structure of each sub-model, and use a graphical notation to represent activation/repression (arrows with non-solid ends) which is introduced in Figure 1. In our modeling, we consider both transcription and translation of proteins. For each species involved in a synthesis process (i.e., transcripts and proteins), the model includes a degradation reaction, not shown in the model diagrams for clarity.

### 2.1. Input signal sub-model

**Figure 2:**
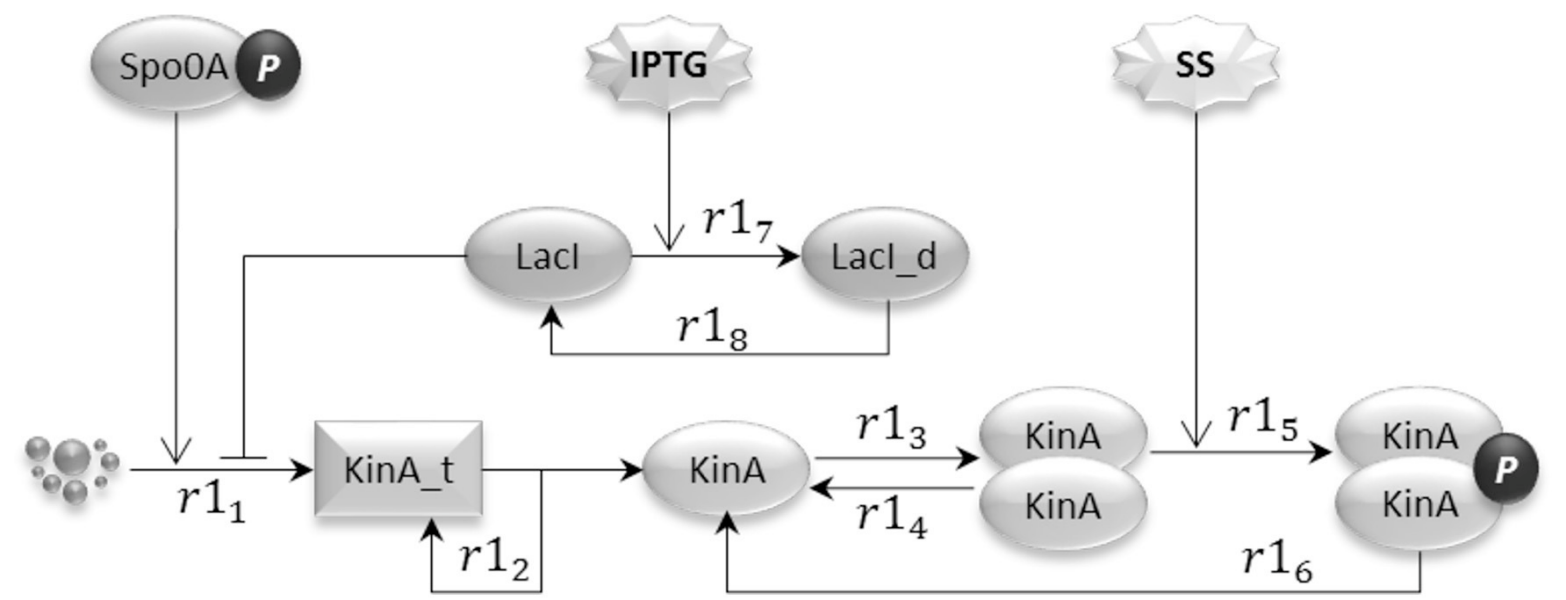
**The input signal model represents the artificial sporulation initiation induced by IPTG through the effect on KinA, as well as the activation of KinA dimers by unknown sporulation signals (SS) generated under unfavorable environmental conditions**.

The input signal sub-model, shown in Figure 2, represents the regulation effects that artificial inducers (in this case IPTG) and cell produced sporulation signals (modeled by species SS) have on the HKs. In the model, reactions are consecutively numbered. In our model, of the five known kinases that have been identified as being capable of initiating sporulation in *B. subtilis* (Jiang et al., 2000), we only considered the histidine kinase KinA. This is the major kinase responsible for initiation of sporulation and its overexpression during exponential growth is sufficient to induce entry into sporulation (Fujita and Losick, 2005).

The IPTG regulation of sporulation is rendered by the indirect release of inhibition for the transcription initiation of KinA (Eswaramoorthy et al., 2009; Narula et al., 2012), exerted by the lactose repressor (LacI) on the binding site incorporated into the promoter. The addition of IPTG causes a conformational change in the LacI protein, bringing it (reaction *r*1_7_) to an inactive form (LacI_d) that has very low affinity for the KinA promoter. The consequence of the inhibition release is an increased level of KinA transcription (reaction *r*1_1_). In the diagrammatic representation, we use a “droplet” notation for species that are not explicitly represented, such as genes in transcription reactions. The LacI conformational change is however reversible (see reaction *r*1_8_). Molecules of KinA transcript are translated into protein molecules (reaction *r*1_2_), which can reversibly bind (reactions *r*1_3_ and *r*1_4_) to form dimers. KinA dimers have the ability to autophosphorylate (reaction *r*1_5_), producing the active species that initiates the phosphorelay signaling (Wang et al., 2001; Eswaramoorthy et al., 2009). We model the dephosphorylation and the unbinding of the KinA dimer as a single reaction (*r*1_6_). The model also considers the activation of the histidine kinase caused by the naturally occurring sporulation signals (SS), which accelerate the KinA autophosphorylation reaction and can lead *B. subtilis* into sporulation alone. Last, the model includes the positive effect that active Spo0A has on the transcription of KinA, via the double repression feedback loop that links phosphorylated Spo0A with AbrB, and AbrB with KinA.

### 2.2. Phosphorelay sub-model

**Figure 3:**
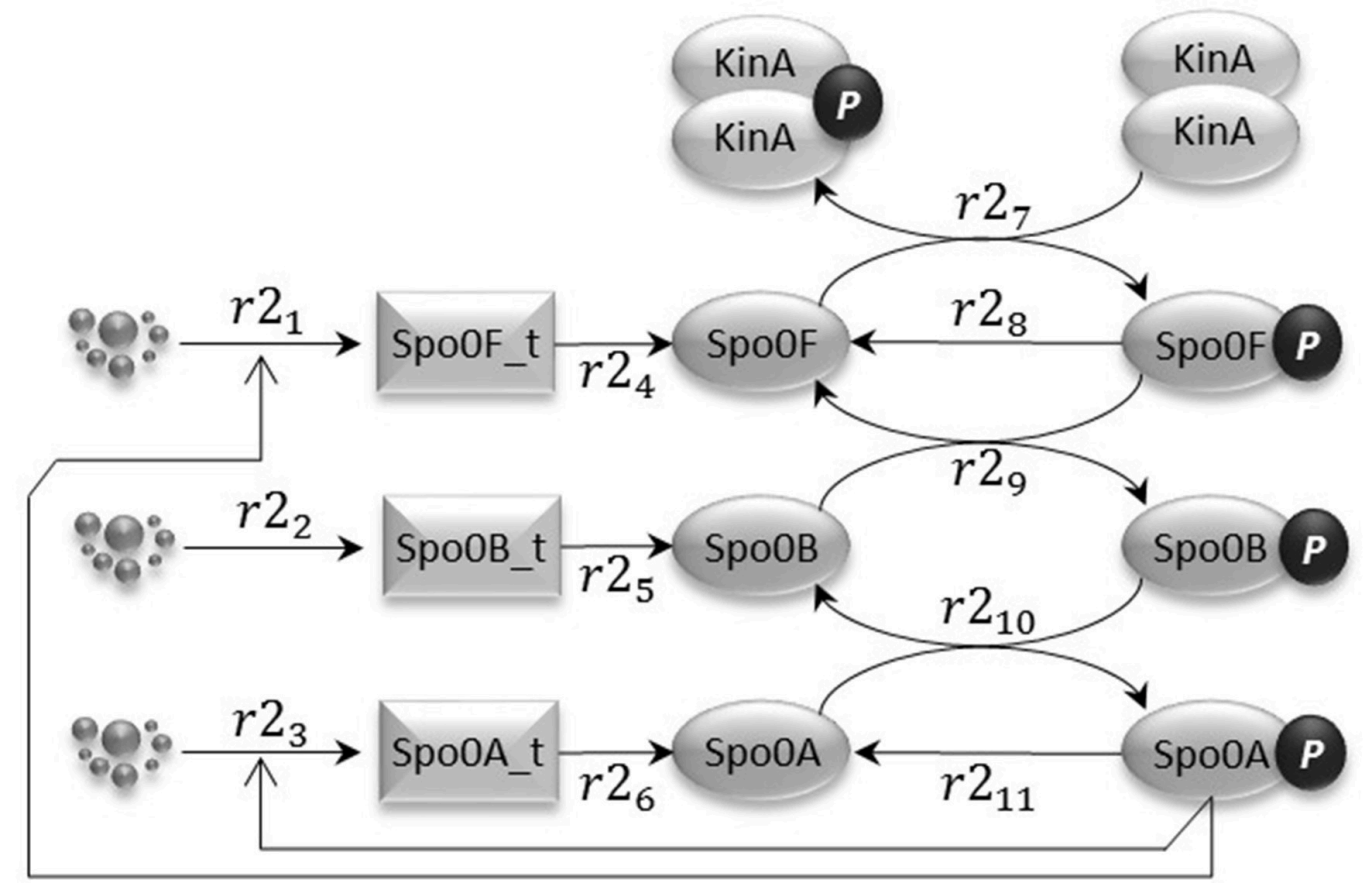
**The phosphorelay sub-model encodes the transfer of phospho groups from activated KinA to Spo0F, which then leads to Spo0A phosphorylation via the phosphotransferase Spo0B**. Dephosphorylation of Spo0F and Spo0A is modeled by abstracting the phosphatase species.

The phosphorelay sub-model depicted in Figure 3 is based on phosphorylation, dephosphorylation and phosphotransfer reactions. Our model includes the main phosphorelay species Spo0F, Spo0B, and Spo0A, which together form a cascading phosphotransfer (de Jong et al., 2010; Sen et al., 2011). For each of these proteins, the model includes a gene transcription reaction (*r*2_1_, *r*2_2_, and *r*2_3_), and a translation reaction (*r*2_4_, *r*2_5_, and *r*2_6_). The phosphorylated KinA dimer transfers the phosphate group to Spo0F (reaction *r*2_7_), phosphorylated Spo0F transfers the phosphate group to Spo0B (reaction *r*2_9_), and finally phosphorylated Spo0B transfers the phosphate group to Spo0A (reaction *r*2_1_0). In the model, phosphorylated Spo0F and phosphorylated Spo0A spontaneously lose the phosphate group (reactions *r*2_8_ and *r*2_11_). Finally, we include in the model the phosphorelay self-activation loop induced by phosphorylated Spo0A, which as already described positively affects the transcription of both Spo0F and Spo0A species.

### 2.3. Gene expression sub-model

**Figure 4:**
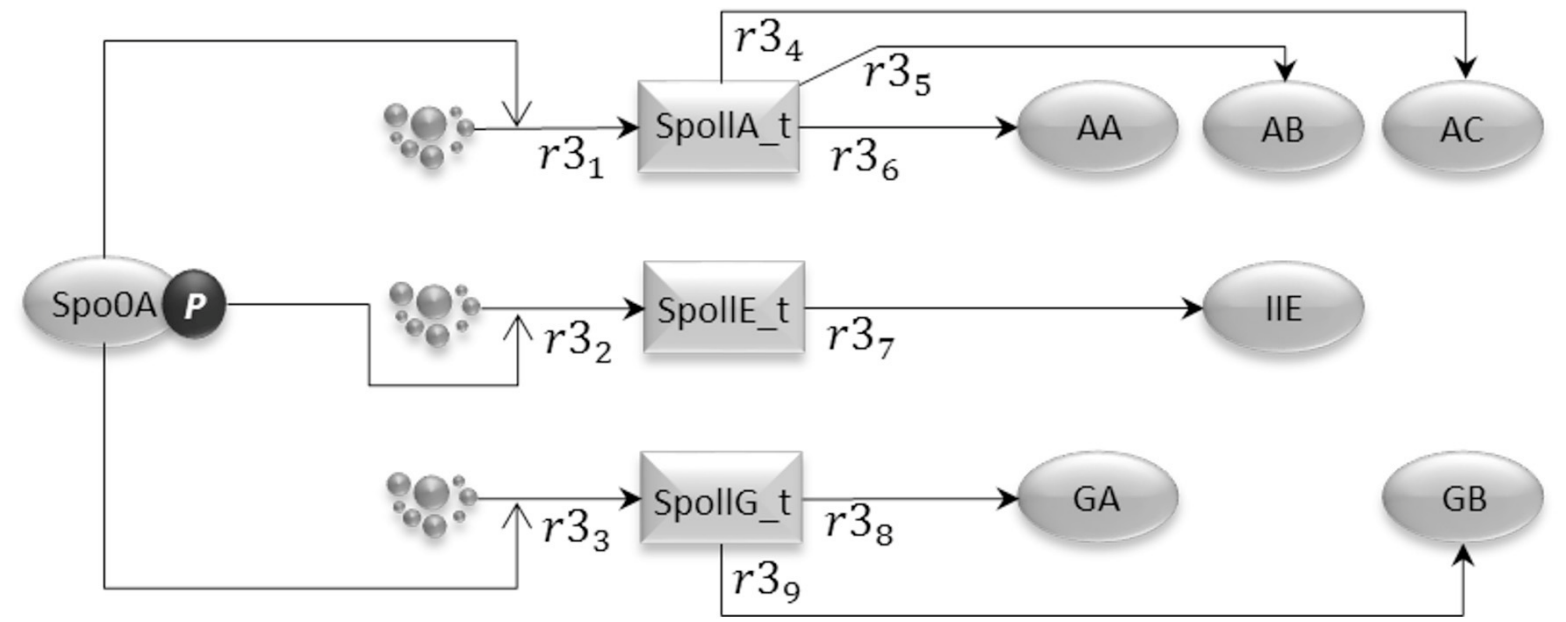
**The gene expression model represents the transcriptional activity of phosphorylated Spo0A, which binds to the Spoll promoters and promotes transcription initiation for the important sporulation initiation proteins, AA, AB, AC, IIE, GA, and GB**.

Phosphorylated Spo0A up-regulates transcription from spoIIA, spoIIE, and spoIIG promoters. The gene expression sub-model shown in Figure 4 encodes the activation of transcription exerted by Spo0A, and includes transcription reactions (*r*3_1_, *r*3_2_, and *r*3_3_) and translation reactions for AA, AB, and AC proteins (*r*3_4_, *r*3_5_, and *r*3_6_), IIE protein (*r*3_7_) and GA and GB protein molecules (*r*3_8_ and *r*3_9_). Notice that AA, AB, and AC, and also GA and GB, are transcribed polycistronically from the spoIIA and spoIIG operons, respectively (Narula et al., 2012).

In the rest of the paper and in the Supplementary Material, we adopt the following notation to indicate the variables corresponding with the molecular species of the model: the name of the protein is written in lowercase (e.g., Spo0A, *spo0a*), the transcript of the gene is denoted by the suffix “_t” (e.g., the transcript of gene Spo0A is denoted by *spo0a_t*), and the phosphorylated form of the protein is indicated by the suffix “p” (e.g., the phosphorilated form of protein Spo0A is *spo0ap*). The mathematical specification of the model and its parameters are given in Tables S1–S4. The time is measured in seconds (s), and the molecular species concentration in nM.

## 3. Detecting chaos in *B. subtilis* sporulation network dynamics

A system is affected by deterministic chaos if its dynamics is governed by deterministic rules and any change in the initial state, no matter how small, grows quickly with time, rendering long-term prediction of the system behavior impossible. A system is affected by *low-dimensional* chaos if only a small number of variables exhibits a chaotic dynamics, i.e., an aperiodic irregular time-behavior (Tél and Gruiz, 2005; Layek, 2015).

The presence of low-dimensional chaos in biological systems is of particular interest, because it indicates that the variables governing the spatial and temporal behavior of the system may be few in number. This means that the dynamics of the system might be controlled by only a few crucial variables. The complexity of control inherent in chaotic systems may be important in the dynamics of gene expression regulation. Therefore, it is of particular interest to assess the presence of low-dimensional chaos in a complex system such as the *B. subtilis* sporulation network, as this analysis allows the identification of the variables (few in numbers) that control the predictability of the dynamics of the whole system.

There are two-established methods to explore chaotic behavior of a dynamical biological system. The first one is a direct analysis of the experimental time series, combined with the development of algorithms for computing relevant indices quantifying the features of the system dynamics. The second is the implementation of a model developed directly from the experimental observations that aims to account for the essential mechanisms at work in the real system and explain the dominant behavior. Then, a subsequent analysis focuses on the simulated time series obtained by model solution and its phase space in order (i) to evaluate the control parameters, (ii) to detect the system components (e.g., genes, proteins, chemical species, etc.) that exhibit a chaotic dynamics, and (iii) to investigate the robustness of the dynamics against perturbations. We implemented the second approach, because the inclusion in the analysis of a model of the systems dynamics affords not only the identification of the drivers of chaotic dynamics, but also the conceptualization of their role and of their effects within the mechanisms of interaction with other molecular species. In the next section, we provide a detailed explanation of this analysis.

### 3.1. Sensitivity analysis

We undertook local sensitivity analysis to assess the sensitivity of the solutions of the system's equations to the variation of individual parameter in the system. We discussed the feasibility of global sensitivity analysis of the system to variation in parameters in the Supplementary Information.

We randomly sampled *N*_*P*_ values from a uniform distribution for each parameter (i.e., kinetic rate constant). The uniform distributions were positively defined on the maximal range of parameter variability in which the system of ordinary differential equations has a unique solution (i.e., it is not underdetermined). We determined this range $I_{q^{*}}\left(p_{h}\right)$ by attempting to solve the system of equations for different sets of parameters *P*(*q*) = {*p*_*h*_}, *h* = 1, 2, …, *N*_*P*_, obtained by varying the value of *q* ∈ ℕ^+^ in the interval [2, 30] in the following expression

(1) $$I_{q}(p_{h})=\left[\frac{p_{h}}{q},q\times p_{h}\right],\;h=1,2,\ldots ,N_{P}$$

The maximal interval of parameter variation is defined by the maximal value of *q* for which the system of differential equations has a unique solution.

The parameters were changed one at a time while keeping the values of the others fixed. Since for each parameter *p*_*h*_ we sampled *N*_*P*_ values, we performed *N*_*P*_ model simulations, i.e., one simulation for each sampled value in the range of parameter variability *I*_*q*_(*p*_*h*_). The index of sensitivity of the time series *x*_*s*_(*t*), (*s* = 1, 2, …, *d*, where *d* is the number of molecular species in the system), with respect to the change of the *h*-th parameter from the value *p*_*h*_ to the value $p_{h}^{\prime }$ is calculated as the mean of the standard deviations of the distributions of the simulated values of the variable over the range of parameter variability and over time, i.e.:

(2) $$\begin{array}{l} SI_{s,h}=\frac{1}{N}\sum_{k\;=\;1}^{N}(\frac{1}{N_{P}-1}\sum_{r\;=\;1}^{N_{P}}(x_{s}^{\left(r\right)}(t_{k}|p_{h}\leftarrow p_{h}^{\prime })-{\bar{x_{s}}}^{\left(r\right)}\\ \;{\;(p_{h}\leftarrow p_{h}^{\prime }){)}^{2})}^{\frac{1}{2}} \end{array}$$

where *N* is the length of the time series, and

(3) $$\begin{array}{l} \bar{x_{s}}\left(p_{h}\leftarrow p_{h}^{\prime }\right)\;=\;\frac{1}{N_{P}}\overset{N_{P}}{\underset{k=1}{\sum }}x_{s}\left(t_{k}|p_{h}\leftarrow p_{h}^{\prime }\right). \end{array}$$

With the expression $p_{h}\leftarrow p_{h}^{\prime }$, we denote the replacement of value *p*_*h*_ with the value $p_{h}^{\prime }$.

### 3.2. Complexity indices

In order to detect the presence of chaos in *B. subtilis* network dynamics, for the time series of each molecular species we calculated a set of indices that capture different aspects of the complexity. This set of indices includes:

1. **Lyapunov exponents (**λ**):** they measure the rate of separation of infinitesimally close trajectories in the phase space generated by slightly different values in the initial state of the system. The largest Lyapunov exponent is usually considered important in the determination of chaotic behavior. A positive value for the largest Lyapunov exponent indicate orbital instability and chaos (Kaneko and Tsuda, 2001; Sprott, 2003; Kalitin, 2004).
2. **fractal dimension (***D*_*F*_**):** a statistical index for pairwise distances of the points of a time series; it indicates how a set of points fills its space and thus quantifies the complexity of the behavior of a trajectory;
3. **sample entropy (***S*_*E*_**):** a measure of data regularity; a smaller value of sample entropy indicates more self-similarity in the data of the time series and a less noise (less disorder);
4. **time lag (***T*_*L*_**):** the time after which the auto-correlation of the time series is negligible;
5. **embedding dimension (***D*_*E*_**):** similarly to the fractal dimension, it measures topological complexity of a time series. A set of points has embedding dimension *D*_*E*_ if *D*_*E*_ is the smallest integer for which it can be embedded into ${\text{R}}^{D_{E}}$ without intersecting itself. So, *D*_*E*_ is the minimum dimension of a space in which a trajectory in the phase space reconstructed from the observed time series does not cross itself (in this case the dynamics is deterministic) (Abarbanel, 1996; Tamma and Khubchandani, 2016).

In chaotic systems, small differences in the initial condition result in strongly different solutions. Therefore, a chaotic system is unpredictable in the sense that the variability of the prediction induced by small changes in the initial conditions is unacceptably high in comparison to the difference of the initial states.

In deterministic systems, complete knowledge of the rules of the dynamics and of the initial state (i.e., values for the abundance of the system's components at initial time *t*_0_–sometimes called *initial conditions*) **x**(*t*_0_), is sufficient to determine **x**(*t*) at each *t* > *t*_0_. In chaotic deterministic systems, if the initial state is changed by a small value **ϵ**, two trajectories that were initially close, will exponentially separate. Formally, if **x**(*t*) and **x**′(*t*) are the two trajectories generated by the initial states **x**(*t*_0_) and ${\text{x}}^{\prime }\left(t_{0}\right)$, and if $|\text{x}\left(t_{0}\right)-{\text{x}}^{\prime }\left(t_{0}\right)|<\epsilon$, we have that

(4) $$\begin{array}{l} |\text{x}\left(t\right)-{\text{x}}^{\prime }\left(t\right)|\sim \epsilon e^{\lambda t} \end{array}$$

where λ is the angular coefficient of the straight line defining ln |**x**(*t*) − **x**′(*t*)| as a function of time *t*:

$$\ln\;|\text{x}\left(t\right)-{\text{x}}^{\prime }\left(t\right)|=\lambda t+\ln\;\epsilon .$$

Using Equation (4) it is possible to predict the time t^*^ after which the predicted trajectory is too imprecise. Indeed, if δ is the tolerance on the precision of the prediction, then from Equation (4) ϵ*e*^λt*^ ~ δ, and therefore

(5) $$\begin{array}{l} t^{*}\sim \frac{1}{\lambda }\ln\;\frac{\delta }{\epsilon }. \end{array}$$

The expression in Equation (5) suggests that t^*^ can be arbitrarily increased by decreasing **ϵ**. However, de facto, it is not possible to obtain a value of t^*^ much greater than $\frac{1}{\lambda }$. For instance, if we want to increase t^*^ by one order of magnitude, we have to decrease **ϵ** by a factor *e*^10^ ~ 10^4^. This example points out that the dependence of t^*^ on the ratio $\frac{\delta }{\epsilon }$ is so weak that in Equation (5), the only term that strongly influences t^*^ is λ (Vulpiani, 2004; Cencini et al., 2009; Lecca et al., 2016).

The system of differential equations describing the dynamics of the *B. subtilis* sporulation network is a *d*-dimensional system, where *d* is the number of molecular species involved in the system. At each instant of time *t* the system is contained in a *d*-dimensional sphere in the phase-space. In particular, this *d*-dimensional sphere is centered at **x**(0) (**x**(0) belonging to the attractor) and has radius ϵ. The time evolution of the system dictated by the equations deforms the sphere into an ellipse. If *l*_*i*_(*t*) denotes the length of the i-th semi-axis of the ellipse at time *t*, the characteristic *Lyapunov exponents* (λ_1_ ≥ λ_2_ ≥ ⋯ ≥ λ_*d*_) are defined as follows:

(6) $$\begin{array}{l} \lambda_{i}=\frac{1}{t}\ln\;\frac{l_{i}\left(t\right)}{\epsilon },\;i=1,2,\ldots ,d. \end{array}$$

If λ_*i*_ > 0, the i-th semi-axis grows with time; in contrast, if λ_*i*_ < 0 the i-th semi-axis shrinks with time. In a system extremely sensitive to the initial conditions, at least one of the Lyapunov exponents is greater than zero.

For each molecular species *i* in the *B. subtilis* network we have calculated the maximal Lyapunov exponent from the corresponding simulated time series, i.e., the Lyapunov exponent at the maximum observed time, formally defined as follows

(7) $$\begin{array}{l} \lambda_{i}^{\text{max}}=\underset{t\to \infty }{\lim}\underset{\epsilon \to 0}{\lim}\lambda_{i} \end{array}$$

The greater a positive maximal Lyapunov exponent, the faster the rate of divergence of the two trajectories **x**(*t*) and **x**′(*t*). Thus, the Lyapunov coefficients were used to measure the contribution of each molecular species to the system's dynamics. In this study, we used the Rosenstein et al. (1993) algorithm to estimate the maximal Lyapunov exponent.

The Lyapunov exponents capture the unpredictability in a system's evolution which can be generated by slightly different initial states. However, unpredictability could depend also on an irregular aperiodic behavior of the abundance of some molecular species in the system.

To capture this aspect of a chaotic dynamical system, and, most importantly to distinguish it from noise, we have estimated the *fractal* and the *embedding* dimensions of the time series of each gene and protein in the system. Both fractal and embedding dimensions are generalizations of the topological dimension and measure the dimensionality of the space occupied by the set of points of the time series. The more complex and irregular the distribution of the points in space is, the higher the fractal and embedding dimensions of the system.

We estimate the fractal dimension as a *correlation dimension* (Theiler, 1990; Ding et al., 1993), defined in terms of the correlation integral *C*(ϵ):

(8) $$\begin{array}{l} C\left(\epsilon \right)=\underset{N\to \infty }{\lim}\frac{g_{\epsilon }}{N^{2}} \end{array}$$

where *N* is the number of points in the time series, and *g* is the total number of pairs of points that dist from each other is less than ϵ (a graphical representation of such close pairs is the *recurrence plot* (Marwan et al., 2016). The correlation integral estimates the probability that a pair of points of the time series is separated by a distance less than ϵ. For ϵ << 1 it can be shown (Theiler, 1990) that

(9) $$\begin{array}{l} C\left(\epsilon \right)\sim \epsilon^{D_{F}} \end{array}$$

where *D*_*F*_ is the correlation dimension. For a sufficiently large, and evenly distributed, number of points in a time series, a log-log graph of the correlation integral vs. ϵ can be used to estimate *D*_*F*_ (Kantz, 2004). The more complex and irregular a time series is, the higher its correlation dimension, as the number of ways for points to be close to each other is greater (Higuchi, 1988). Indeed the fractal dimension corresponds to the number of the degrees of freedom of the time series (Mera and Morán, 2002).

Unlike topological dimension, the fractal dimension can take non-integer values, indicating that a set of points of a trajectory can fill its space qualitatively, and quantitatively, in a different way from an ordinary geometrical set. For instance, a curve with fractal dimension very near to 1, behaves quite like an ordinary line, but a curve with fractal dimension greater than 2 winds convolutedly through space very nearly like a surface or a volume. As a consequence, if a time series of a gene or protein has a fractal dimension significantly greater than 1, the dynamics of that gene or protein is more likely affected by chaos than by noise.

The sample entropy *S*_*E*_ adds further information to that provided by the Lyapunov exponents and the fractal dimension, as it is a direct measure of the unpredictability of a time series (Mao, 2011). Indeed, *S*_*E*_ estimates how much a given data point depends on the values of a number *m* of preceding data points, averaged over the whole time series. *S*_*E*_ is computed as the negative logarithm of the conditional probability that two similar samples from the time series remain similar at the next point (Richman and Moorman, 2000; Azar and Vaidyanathan, 2016). To calculate the sample entropy, points matching within a tolerance ϵ are computed until there is no match according to this condition. Formally, if

$$X\left(t\right)=\left\{x\left(t_{1}\right),x\left(t_{2}\right),\ldots ,x\left(t_{N}\right)\right\}\equiv \left\{x_{1},x_{2},\ldots ,x_{N}\right\}$$

is a time series of length *N*, the sample entropy is defined as in the following by Azar and Vaidyanathan (2016), Sokunbi (2014), and Richman and Moorman (2000).

(10) $$\begin{array}{l} S_{E}\left(m,r,N,\tau \right)=-\ln\;\frac{U^{\left(m+1\right)}\left(\epsilon \right)}{U^{\left(m\right)}\left(r\right)} \end{array}$$

where *B*_*i*_ is the number of *j* where |*X*(*i*) − *X*(*j*)| ≤ *r*, and

$$\begin{array}{l} U^{\left(m\right)}\left(\epsilon \right)=\frac{1}{N-m\tau }\overset{N-m\tau }{\underset{i=1}{\sum }}\frac{B_{i}}{N-\left(m+1\right)\tau }\\ X_{m}\left(i\right)=\left\{x_{i},x_{i+\tau },\ldots ,x_{i+\left(m-1\right)\tau }\right\}\\ X_{m}\left(j\right)=\left\{x_{j},x_{j+\tau },\ldots ,x_{j+\left(m-1\right)\tau }\right\}\\ 1\leq j\leq N-m\tau ,\;j\neq i. \end{array}$$

*X*_*m*_(*i*) is called *template vector* of length *m* of the time series *X*(*t*), and an instance where a vector *X*_*m*_(*j*) is within ϵ of *X*_*m*_(*i*) is called a template *match*. The quantity $\frac{B_{i}}{N-\left(m+1\right)\tau }$ is the probability that any vector *X*_*m*_(*j*) is within *r* of *X*_*m*_(*j*). Finally, τ is called *time delay*, and in our analysis it has been set equal to the time lag $T_{L}^{*}$, that is an estimate of the time at which the time series behavior becomes unpredictable. It can be computed using the auto-correlation function method (Zeraoulia, 2011) and is taken as the lag time $T_{L}^{*}$ at which the auto-correlation function

(11) $$\begin{array}{l} r_{T_{L}}=\frac{\overset{N-T_{L}}{\underset{i=1}{\sum }}\left(x_{i}-\bar{x}\right)\left(x_{i+T_{L}}-\bar{x}\right)}{\overset{N}{\underset{i=1}{\sum }}{\left(x_{i}-\bar{x}\right)}^{2}}. \end{array}$$

first crosses zero. This choice of τ in the estimation of sample entropy is motivated by the need to capture also non-linear autocorrelation properties of the time series (Kaffashia et al., 2008). For instance, it has been proved that with a unity time delay (Kaffashia et al., 2008), the sample entropy measures only the linear autocorrelation properties of the time series. A lower value of *S*_*E*_ (and a higher value of $T_{L}^{*}$) indicates higher predictability of the time series, while a higher value of *S*_*E*_ (and a lower value of $T_{L}^{*}$) indicates lower predictability.

Finally, we also considered the embedding dimension as a measure of time series complexity. The embedding dimension of a time series is the smallest dimension required to embed it, and it can be estimated by the Cao's algorithm (Cao, 1997). In our analysis, the parameter *m* in the definition of sample entropy has been set equal to the embedding dimension.

#### 3.2.1. Distinguishing noise from chaos

Since both the presence of chaos and the presence of noise are manifested as topological and statistical complexity of a time series, our analysis aims to distinguish chaos from noise. In the past decade many methods in a different application domains have been proposed to make this distinction, the most recent are reported in Skiadas and Skiadas (2016), Ravetti et al. (2014), Rohde (2008), Gao et al. (2006), and Rosso et al. (2007).

We adopted a simple well established method based on the comparison of the complexity indices identified above and obtained from the time series simulated by the model with those obtained from Gaussian white noise, colored noise and power-law noise (Skiadas and Skiadas, 2016). The expectation is that sample entropy, time lag, and embedding dimension for the non-noisy candidate chaotic times series are significantly different from those estimated for the white and colored noise time series. Moreover, the time behavior of the Lyapunov exponents is expected to be non-linear for the noise and at least linear for chaotic non-noisy time series (Gao and Zheng, 1994).

### 3.3. Analysis of the jacobian matrix: the time evolution of the phase space

In order to explore the phase space of the systems and calculate its equilibria and its time evolution we analyzed the Jacobian matrix **J** of the system of ordinary differential equations describing the dynamics.

(12) $$J=\left[\begin{array}{cccc} \frac{\partial f_{1}}{\partial S_{1}} & \frac{\partial f_{1}}{\partial S_{2}} & \ldots & \frac{\partial f_{1}}{\partial S_{d}}\\ \frac{\partial f_{2}}{\partial S_{1}} & \frac{\partial f_{2}}{\partial S_{2}} & \ldots & \frac{\partial f_{2}}{\partial S_{d}}\\ \ldots & \ldots & \ldots & \ldots \\ \frac{\partial f_{d}}{\partial s_{1}} & \frac{\partial f_{d}}{\partial s_{2}} & \ldots & \frac{\partial f_{d}}{\partial s_{d}} \end{array}\right]$$

where $f_{i}=\frac{ds_{i}}{dt}$, and *s*_*i*_ is the abundance of the i-th molecular species in the system (*i* = 1, 2, …, *d*).

A *steady state* point $s_{eq}=\left\{s_{i}^{eq}\right\}$, *i* = 1, 2, …, *d*, of the systems is defined by a solution of the system of algebraic equations as in the follows:

$$\frac{ds_{i}}{dt}=0,\;i=1,2,\ldots ,d.$$

The stability of a steady state point is determined by the sign of the real part of the eigenvalues of the Jacobian matrix. In particular, if the real parts of all eigenvalues are negative, the steady state point is stable. It's termed *sink*, because, there is a basin around it, and any initial condition in that basin will result in a trajectory falling in toward the steady state point.

If the real parts of all the eigenvalues are positive, the steady state point is *unstable*. It is termed *source*, because, starting from an initial point close to it, the trajectory will move away from it. If the real parts of the eigenvalues are of different signs, the steady state point is called a *saddle* point. It is *unstable*, attracting along some axes and repelling along others. If there are also complex components, the nature of the fixed point doesn't change (it's still a sink, source, or saddle point) but with a twist. If the eigenvalues are purely complex, then there are closed orbits around the fixed point.

The eigenvectors of the Jacobian matrix give the axes along which the behaviors indicated by the eigenvalues are centered. So, the eigenvector associated with a negative eigenvalue is a vector along which the fixed point attracts. The eigenvector associated with a positive eigenvalue is an axis along which the fixed point repels.

## 4. Results

In this section we collect the results of three different analyses: (i) the parameter sensitivity analysis, (ii) the model time series analysis, and (iii) the phase space analysis. The first two analyses capture different aspects and manifestations of the presence of chaos and their outputs are sets of molecular species whose behavior is a likely candidate for chaotic dynamics. The final result is an intersecting set of molecular species that represents the consensus set of molecular species whose dynamics is affected by chaos. The third analysis aims at determining how the topology and the parameters of the network of interactions among the molecular species evolves with time. This last analysis allows the determination of the time variation of the active degrees of freedom in the system (Hilborn, 2000).

### 4.1. Kinetic rate constants controlling the dynamics

**Figure 5:**
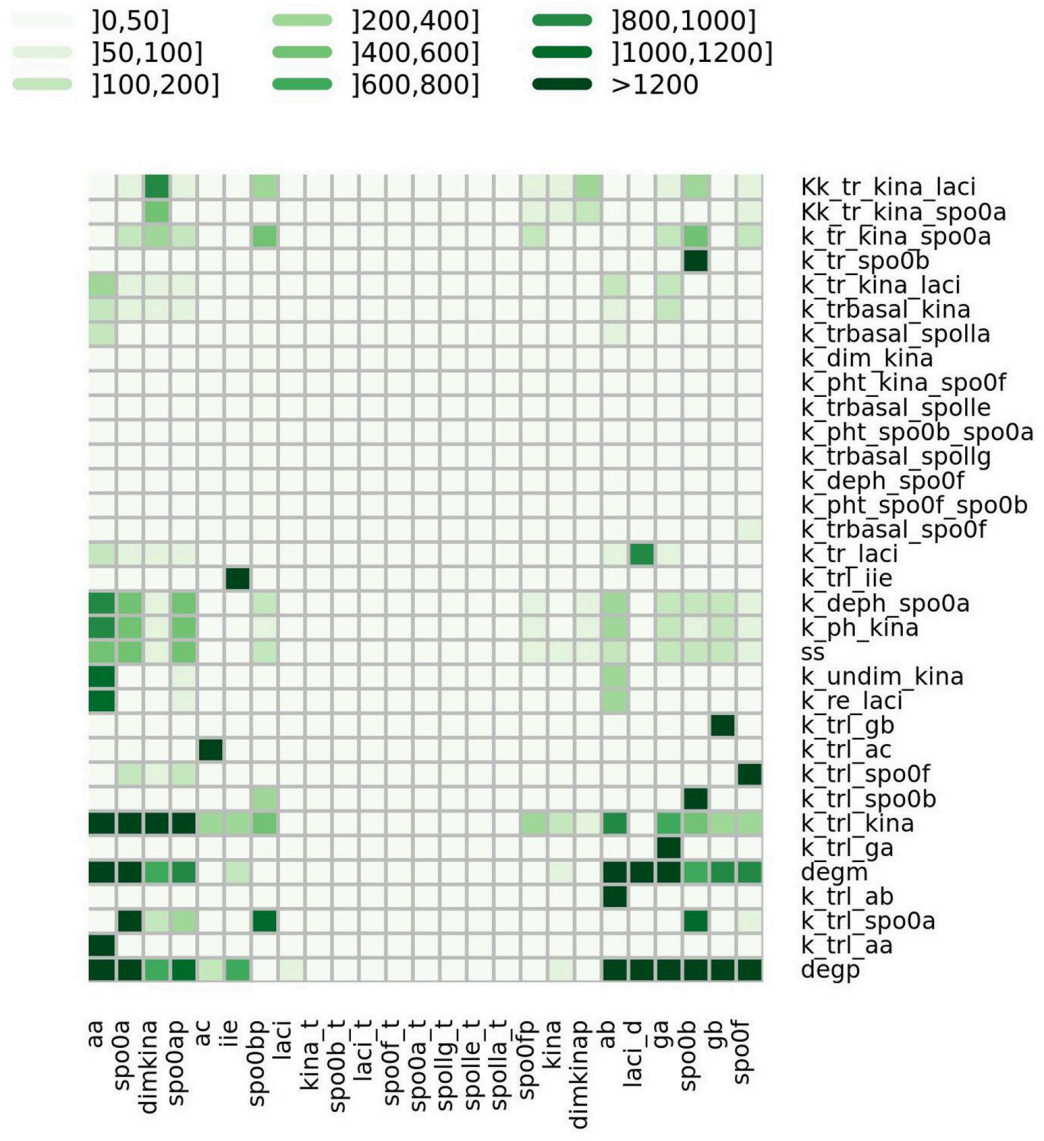
**Heatmap summarizing the results of sensitivity analysis**. The color of the cell is indicative of the value of the sensitivity index *S* as given in Equation (2), categorized by intervals.

We explored the parameters' space in which the systems of ordinary differential equations that represent the model has a solution. We found that the largest range of variation for the parameters at which the systems still admits a unique solution is defined by

(13) $$I(p_{h})=\left[\frac{p_{h}}{10},10\times p_{h}\right],\;h=1,2,\cdots ,N_{P}$$

where *p*_*h*_ is the value of the h-th parameter assigned from experimental data. For each parameter we randomly sampled 50 values from a uniform distribution positively defined in *I*(*p*_*h*_). In turn these were used in simulations to give sensitivity indices according to Equation (2). In Figure 5 a heatmap shows the value of the sensitivity index collapsed into intervals. Moreover, Table 1 lists the variables and the parameters which affect them most. Appreciable parameter sensitivity is only apparent in an interval ranging from a tenth to ten times the parameter value obtained from data. The dynamics of the majority of the molecular species is robust with respect to the variations of the parameters' values on smaller intervals (see Figure S2). The molecular species *spo0a, spo0b, spo0ap*, and *spo0bp* are the most sensitive to perturbation of parameters even on small intervals.

**Table 1:** **Molecular species and the parameters mostly controlling their dynamics**.

**Molecular species**	**Parameters**
*aa*	degp,k_trl_aa
*spo0a*	degp,k_trl_spo0a
*dimkina*	k_trl_kina,Kk_tr_kina_laci
*spo0ap*	k_trl_kina,degp
*ac*	k_trl_ac,k_trl_kina
*iie*	k_trl_iie,degp
*spo0bp*	k_trl_spo0a,k_trl_kina
*ab*	degp,k_trl_ab
*laci_d*	degp,degm
*ga*	degp,k_trl_ga
*spo0b*	degp,k_trl_spo0b
*gb*	degp,k_trl_gb
*spo0f*	degp, k_trl_spo0f

### 4.2. Complexity indices identify the drivers of chaotic dynamics

**Figure 6:**
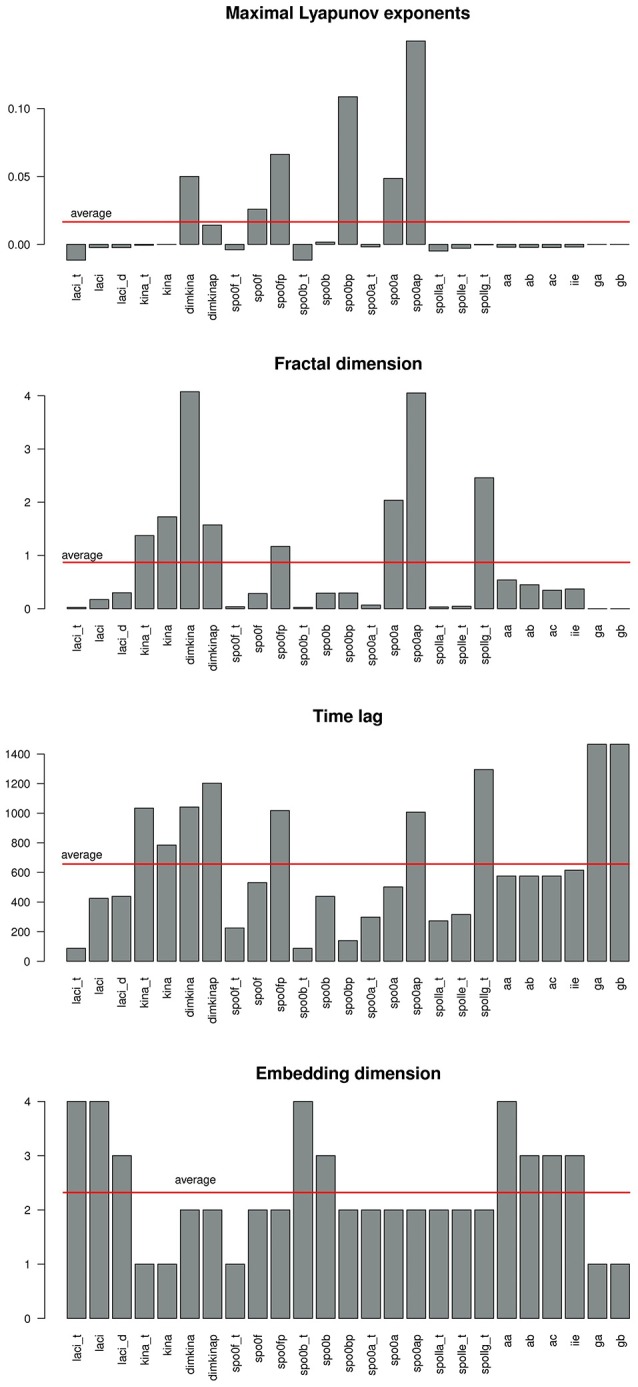
**Barplot showing the values of the complexity indices maximal Lyaopunov exponent (A), fractal dimension (B), time lag (C), and embedding dimension (D) estimated from the time series of each molecular species in the system**. A red line marks the average value.

**Figure 7:**
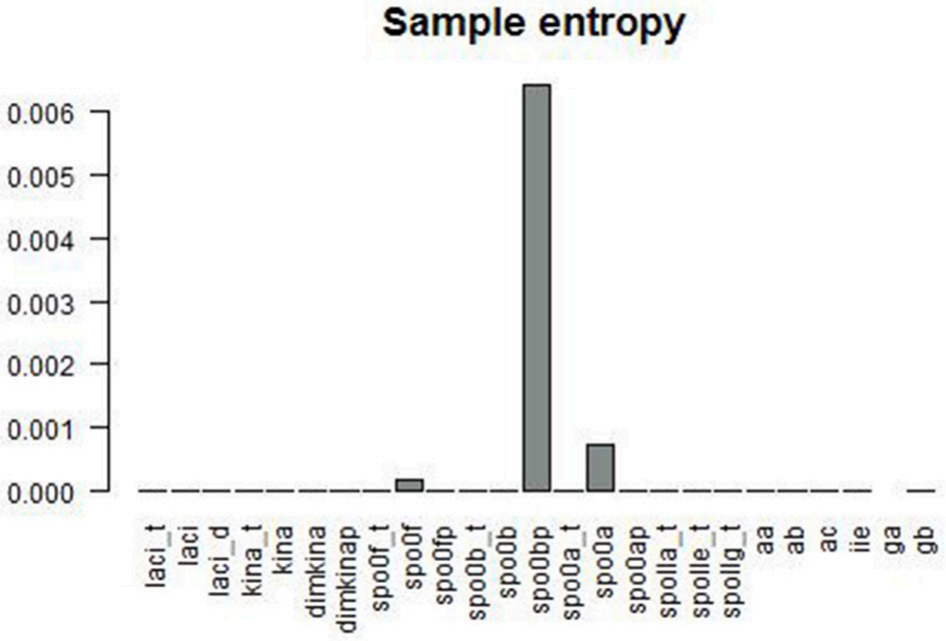
**Sample entropy is a measure the repeatability or predictability within a time series**. *spo0bp* has a sample entropy about six times greater than the sample entropy of the other molecular species.

Figure 6 gives a graphical summary of the complexity indices estimated for the time series of each molecular species. The majority of the molecular species have positive Lyapunov exponents, fractional dimension, time lag between 0 and 400 (that is the about 3% of the time range used in the simulation), and embedding dimension greater than 1. Figure 7 shows the sample entropy values and reports that the highest vale of sample entropy is assumed by spo0bp.

**Figure 8:**
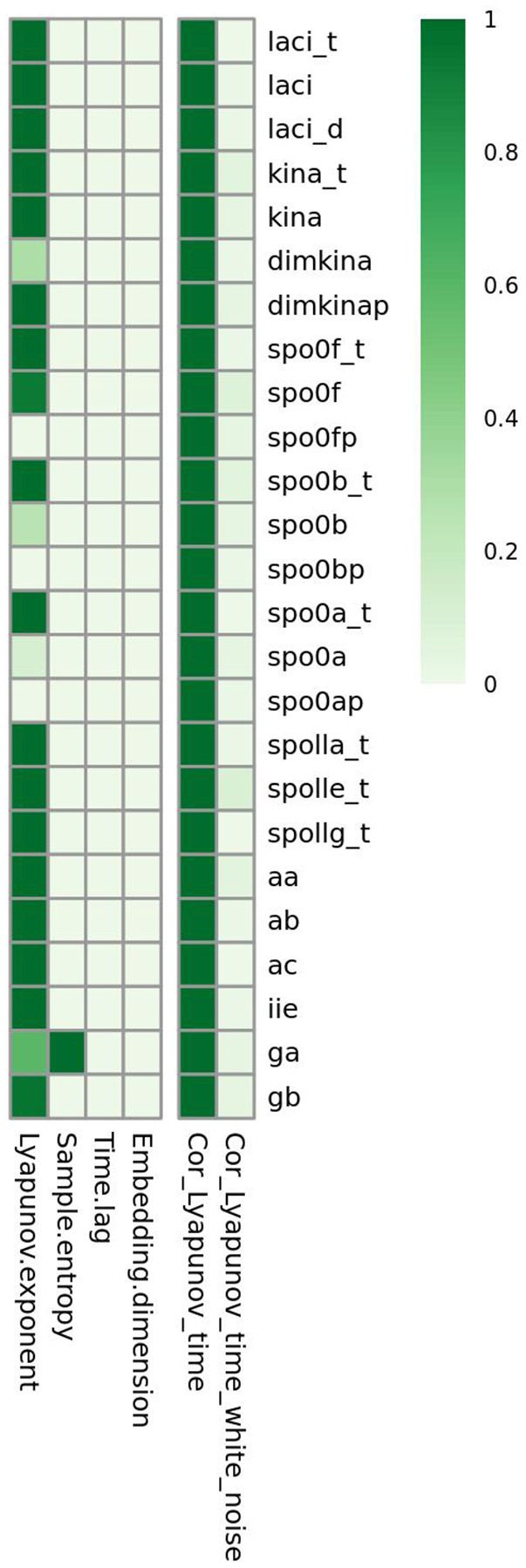
**Complexity detected in the *B. subtilis* sporulation network dynamics is due to low dimensional chaos and not to noise**. For the time series of molecular species *spo0a, spo0b, spo0ap, spo0bp, spo0f*, and *spo0fp*, all the indices of complexity are significantly greater than those for the time series of Gaussian white noise.

To distinguish chaotic from noisy dynamics, we compared the complexity indices of the time series of each variable with the mean and the standard deviation of the complexity indices estimated from 50 time series of white Gaussian noise of mean μ = 0, variance σ^2^ = 1 generated for each variable and having amplitude equal to the range of variability of the variable. The heatmap in Figure 8 shows, on the left side, the frequency at which the noisy time series has an index value higher than that observed for the time series from the real model. Comparison of indices under chaotic and noisy conditions is performed for each index (shown by column) and each variable (shown by row). The heatmap on the right side shows correlation between Lyapunov index and time points in the time series. Column “Cor_Lyapunov_time” displays statistical significance for Pearson's correlation relating Lyapunov exponent and time. Column “Cor_Lyapunov_time_white_noise” displays similar information for the time series of white Gaussian noise generated for each variable. We found that sample entropy, time lag and embedding dimension are significantly higher in the model time series than in the white noise time series. The Lyapunov exponents are significantly greater for the white noise time series compared with the model's time series, except for *spo0a, spo0ap, spo0bp, spo0f*, and *spo0fp*. This result suggests that these molecular species exhibit remarkable chaotic dynamics. The left part of the heatmap confirms a non linear time behavior of the Lyapunov exponents of the white noise time series, and suggests a linear time behavior of the Lyapunov exponents of the model's time series. Again, this result distinguishes between chaotic dynamics and random noisy dynamics (Gao and Zheng, 1994). In the Supplementary Material (Figures S4–S6), we report similar results obtained in the comparison of the complexity indices of the model's time series with the ones for the colored and power law noise.

**Figure 9:**
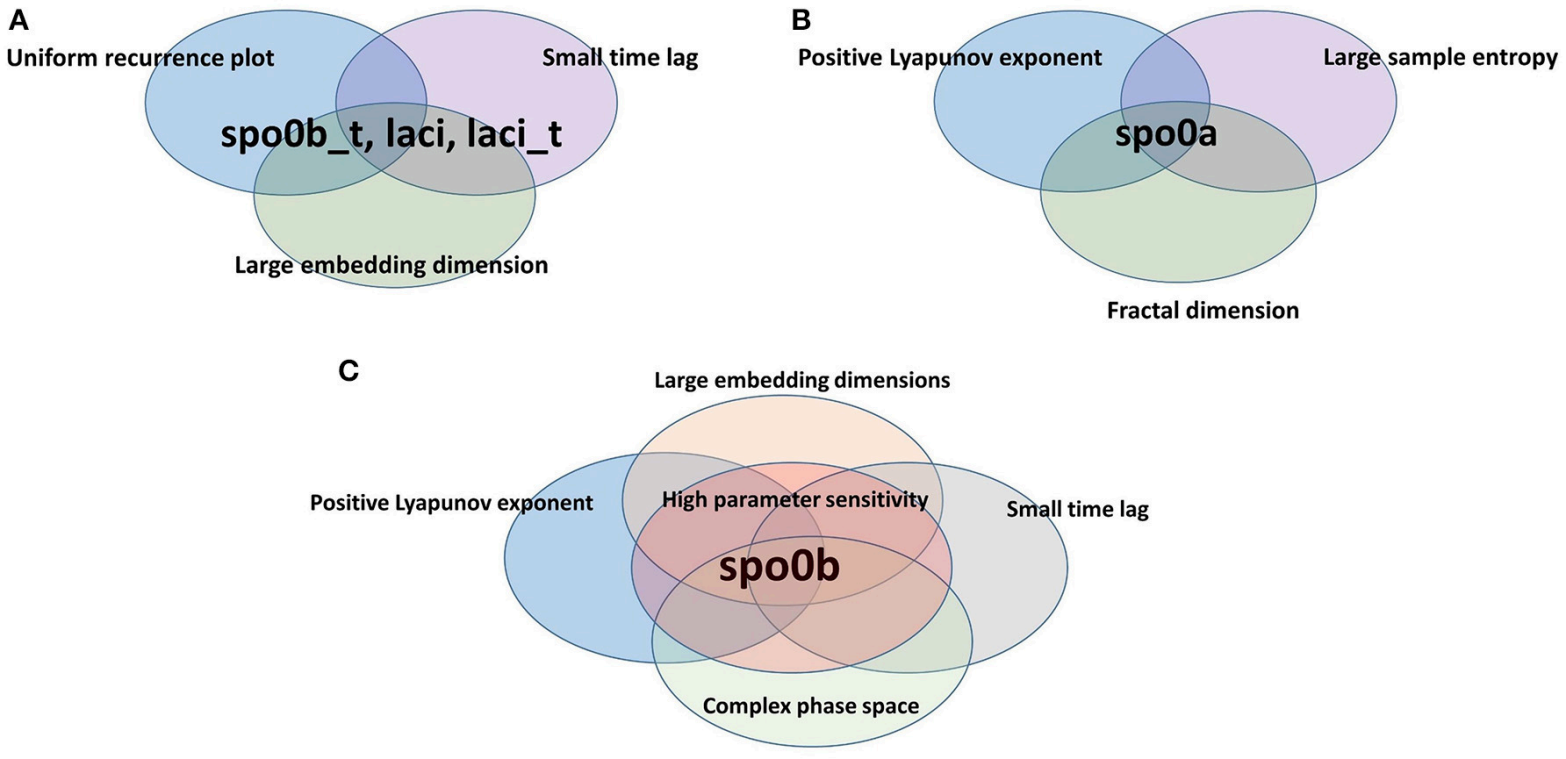
**Species scoring positive to complexity indices**. Species are organized according to the different sets of complexity indices they resulted to be positive to. **(A)** *spo0b_t, laci*, and *laci_t* are characterized by uniformly irregular recurrence plot, a short time lag and an embedding dimension greater than the average. Indeed, they belong to the intersection of the set of species listed on rows 3, 4, and 7 of Table 2. **(B)** *spo0a* has a maximal positive Lyapunov exponent, a large sample entropy and is fractional in dimension. Indeed, it belongs to the intersection of the sets of species listed on rows 1,2, and 5 of Table 2. **(C)** *spo0b* has a maximal positive Lyapunov exponent, an embedding dimension greater than 1, a short time lag, and a high sensitivity to parameters. It belongs to the intersection of sets of species on rows 1, 3, 7, and 8 of Table 2.

For each index of complexity, Table 2 (and a graphical summary of it in Figure 9) reports the set of molecular species where results indicate the presence of chaos. In this Table, we also included two qualitative indicators of the presence of chaos, such as a complex phase space (i.e., convoluted trajectories) and recurrence plot. These plots are provided in Supplementary Material (Figure S7), and visualize a square matrix, whose elements are the times at which a state of a dynamical system recurs (columns and rows correspond then to a certain pair of times) (Marwan et al., 2016, 2007). We refer the reader to the Supplementary Material for a comprehensive description of the recurrence plots analysis.

**Table 2:** **Sets of molecular species with values of the complexity indices indicative of presence of chaos**.

**Index**	**Variables**	**% of total nr. of variables**
Positive Lyapunov exponent	dimkina, dimkinap, spo0f, spo0fp, spo0b, spo0bp, spo0a, spo0ap	32
Fractal dimension (above the average)	kina_t, kina, dimkina, dimkinap, spo0fp, spo0a, spo0ap, spollg_t	32
Embedding dimension (above the average)	kina_t, kina, dimkina, dimkinap, spo0fp, spo0a, spo0ap, spollg_t	36
Time lag (below the average)	laci_t, laci, laci_d, spo0f_t, spo0f, spo0b_t, spo0b, spo0bp, spo0a_t	64
	spo0a, spolla_t, spolle_t, aa, ab, ac, iie	
Sample entropy (above the average)	spo0f, spo0bp, spo0a	12
Complex trajectory in phase space	ga, gb, spo0a, spo0a_t, spo0b, spo0f_t, spollg_t	28
Recurrence plot reveals chaos	spo0b, spo0bp, spo0a, spo0ap, aa, ab	24
High parameter sensitivity (ordered by sensitivity)	aa, spo0a, dimkina, spo0ap, ac, iie, spo0bp, ab, laci_d, ga, spo0b, gb, spo0f	52

The third column reports the percentage of species scoring positive to the test of chaos according to a set of indices.

The variable *spo0b* is the one with the largest set of complexity indices whose values point to the presence of chaos.

### 4.3. Phase space analysis

**Figure 10:**
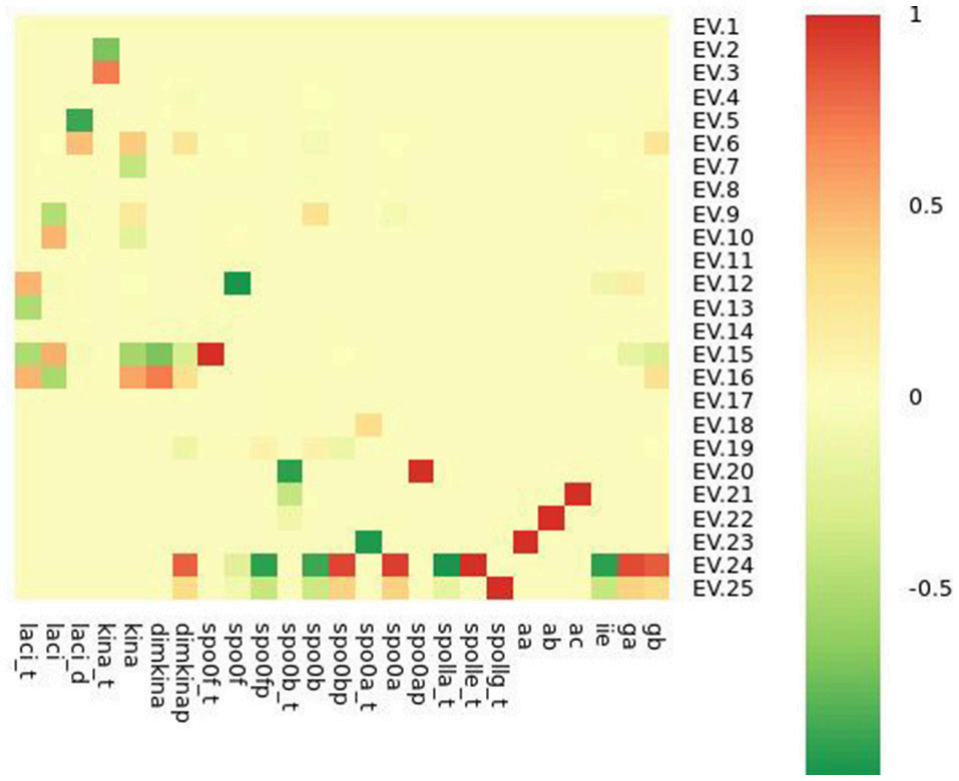
**Heatmap representation of the Jacobian matrix eigenvectors (EVs) evaluated at the steady state point reported in Table 3**.

**Figure 11:**
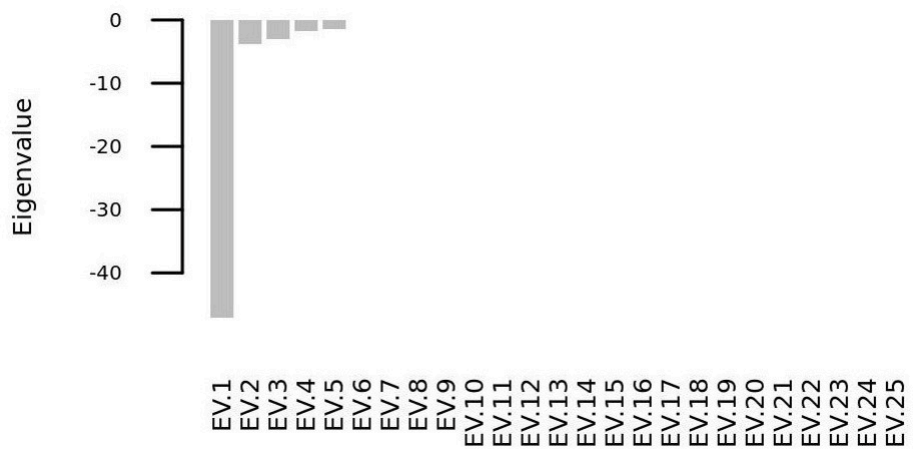
**All the eigenvalues of the Jacobian matrix at the steady state point are negative and thus the steady state point is a stable attractor**.

We solved the systems of differential equations setting to zero the initial concentration of all the molecular species (i. e. *s*_*i*_(*t* = 0) = 0∀*i* = 1, 2, …, 25), and we found that the system has one steady state point, whose coordinates are shown in Table 3. This point is a stable equilibrium, as the eigenvalues of the Jacobian matrix (Figure 10) of the system are all negative (Figure 11).

**Table 3:** **Coordinates of the stable steady state point of the model solved with initial conditions *s*_*i*_(*t* = 0) = 0 ∀*i* = 1, 2, …, 25**.

**Molecular species**	**Coordinate (nM)**
*laci_t*	17.24
*laci*	18.27
*laci_d*	2855.29
*kina_t*	77.89
*kina*	674.32
*dimkina*	1719.72
*dimkinap*	81.81
*spo0f_t*	20.68
*spo0f*	1219.14
*spo0fp*	26.7
*spo0b_t*	41.1
*spo0b*	3683.52
*spo0bp*	2.09
*spo0a_t*	23.92
*spo0a*	2350.24
*spo0ap*	1921.15
*spolla_t*	76.6
*spolle_t*	57.45
*spollg_t*	81.74
*aa*	7979.31
*ab*	3542.82
*ac*	880.92
*iie*	660.73
*ga*	2316.09
*gb*	940.06

We also calculated the value of the elements of the Jacobian matrix at different time points to determine its time evolution. Since the entries of the Jacobian matrix are the partial derivatives of the rate equations with respect to the variables [i.e., $J_{ij}=\frac{\partial f_{i}}{\partial S_{j}}$ (Equation 12)], the Jacobian matrix can be represented by a weighted graph, where the nodes represent the variables (i.e., the molecular species) and the edge weights are the elements of the matrix *J*_*ij*_. We introduced the estimate of the error on *J*_*ij*_ defined as $\Delta J_{ij}=\text{prec}\times \frac{\partial^{2}f_{i}}{\partial S_{j}^{2}}$, where prec = 10^−8^ is the precision of the numerical solution of the model, and set the threshold of 20% on the relative error $ER=\frac{\Delta J_{ij}}{J_{ij}}$. Edges with *ER* < 20% were retained and and allowed for the estimation of the number of active degrees of freedom in the system, i.e., the number of variables involved in active interactions (Hilborn, 2000). Hence, the graphs derived from the Jacobian matrices estimated at different time points are temporal snapshots of the molecular interaction network.

**Figure 12:**
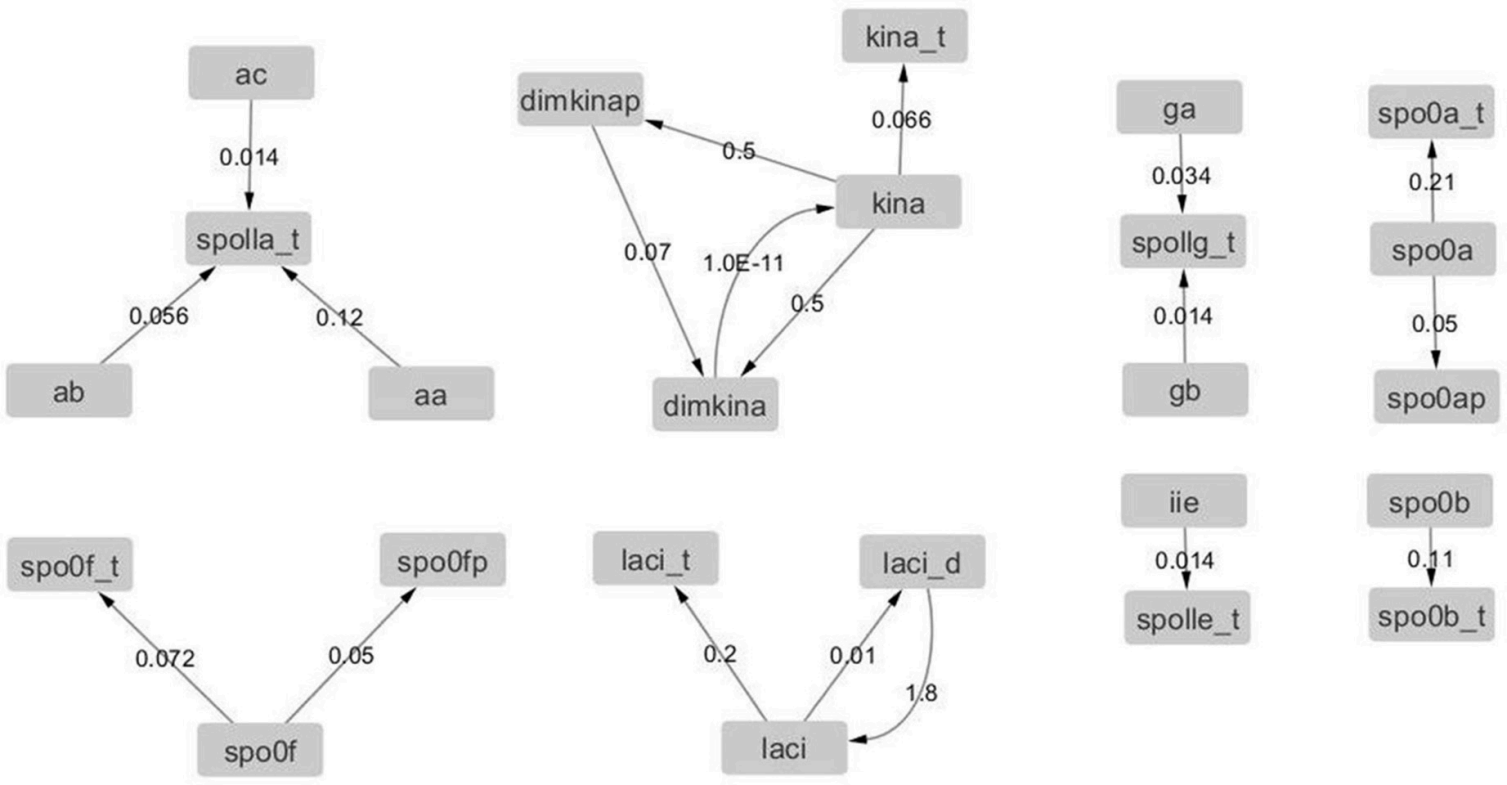
**Graph with adjacency matrix equal to the Jacobian matrix at time *t* = 0 s**. The graphs shows the basal reactions, i.e., those that initiate the time evolution for the network.

**Figure 13:**
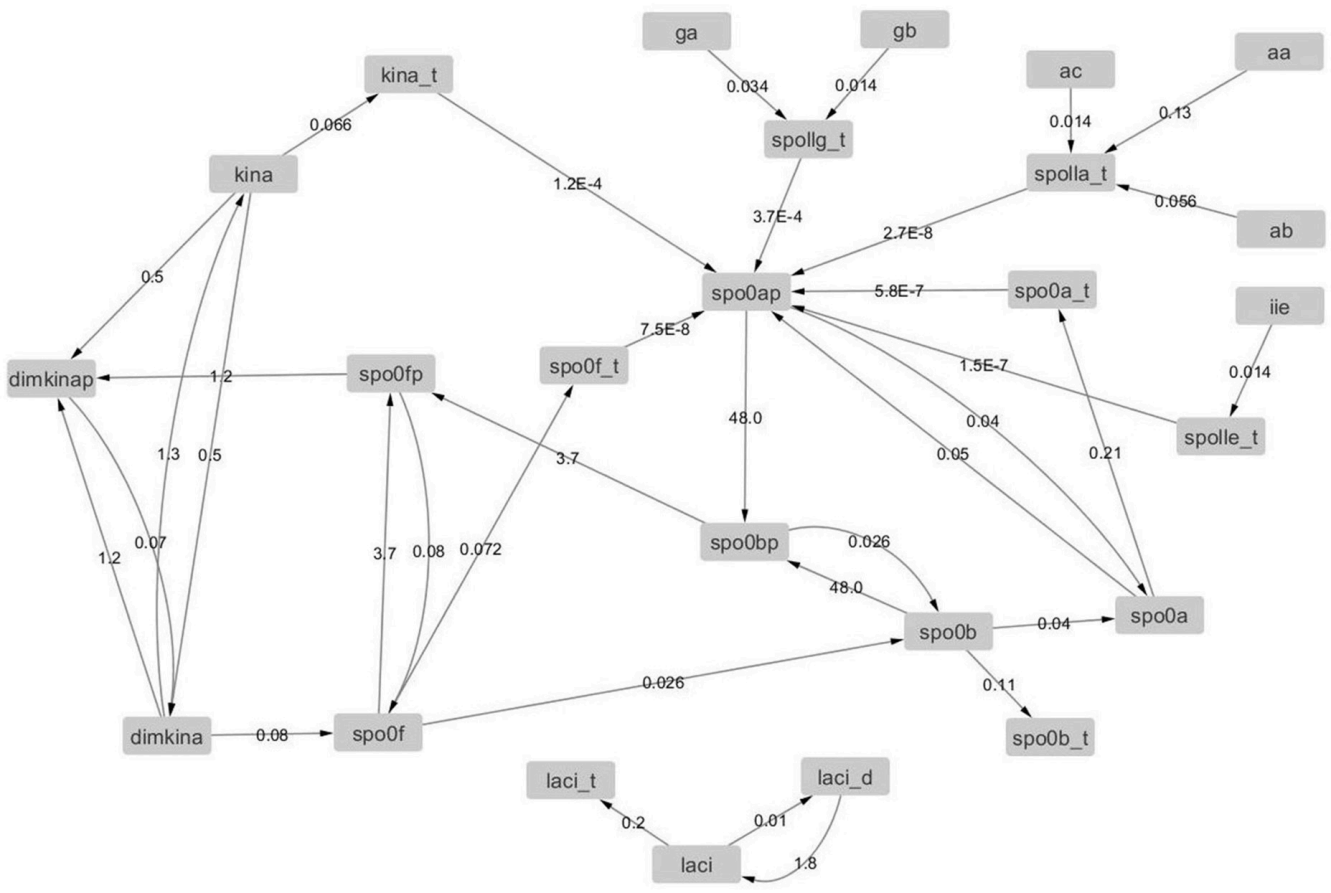
**Graph with adjacency matrix equal to the Jacobian matrix at time *t* = 500**.

The graphs derived from the Jacobian matrix estimated at different time points are time snapshots of the interaction network of the molecular species. These graphs visualize the interactions that are active (i.e., with an edge that has a weight significantly different from zero) at a given time, and thus provide an approximate estimation and representation of the number of active degree of freedom of the system. We report in Figures 12, 13, the graphs obtained from the Jacobian matrices evaluated at times *t* = {0, 500} s. For *t* > 500 s the variations of the Jacobian matrix are minimal and thus not shown here (in the Supplementary Material we provide the graphs for *t* > 500 s in GraphML format).

In Table 4, we observe a rapid increment of the edge weights from *t* = 0 to *t* = 500, than a plateau till *t* = 4, 000, and then a decrement at *t* = 4, 000 s. These changes reflect the changes of the topology of the network of *B. subtilis* sporulation initiation. These steep decrements and increments are expressions of a stiff and highly non linear dynamics, and in turn confirms the presence of chaos (unpredictability) in (of) it.

**Table 4:** **Minimum, first quartile, median, mean, third quartile, and maximum of the distributions of the Jacobian matrix values (i.e., edge weights of the networks) at times *t* = (0, 500, 1000, …, 7500)**.

**Time (sec)**	**Min**.	**1st Qu**.	**Median**	**Mean**	**3rd Qu**.	**Max**.
**0**	**0.00**	**0.02**	**0.07**	**0.20**	**0.16**	**1.80**
500	0.00	0.01	0.07	3.09	0.50	48.00
1000	0.00	0.01	0.07	3.09	0.50	48.00
1500	0.00	0.01	0.07	3.04	0.50	47.00
2000	0.00	0.01	0.07	3.04	0.50	47.00
2500	0.00	0.01	0.07	3.03	0.50	47.00
3000	0.00	0.01	0.07	3.04	0.50	47.00
3500	0.00	0.01	0.07	3.04	0.50	47.00
**4000**	**0.00**	**0.01**	**0.05**	**0.90**	**0.46**	**11.00**
4500	0.00	0.01	0.05	2.76	0.50	43.00
5000	0.00	0.01	0.06	3.30	0.50	52.00
5500	0.00	0.01	0.07	3.36	0.50	53.00
6000	0.00	0.01	0.07	3.25	0.50	51.00
6500	0.00	0.01	0.07	3.20	0.50	50.00
7000	0.00	0.01	0.07	3.14	0.50	49.00
7500	0.00	0.01	0.07	3.09	0.50	48.00

Steep changes of the values of the quartiles reveal an irregular behavior of the dynamics, and reflect the presence of chaos.

#### Sensitivity to initial conditions

In order to assess the existence and, eventually, the sensitivity of the steady state point in response to perturbations of the initial conditions, we introduced a set Δ of perturbations of different magnitudes: Δ = {Δ_*h*_, *h* = 1, 2, …, 7}, where $\Delta_{1}=10^{-5}$ and Δ_*i*+1_ = 10Δ_*i*_, *i* = 1, 2, …, 6. We perturbed the initial state of one variable at a time by Δ_*h*_ (*h* = 1, 2, …, 7), and calculated the steady state point with the Newton-Raphson method (Deuflhard, 2004; Bressoud, 2007), which is one of the most consolidated and efficient algorithms for finding the zeros of a function (Hoppensteadt, 1993; Ortega and Rheinboldt, 2000).

**Figure 14:**
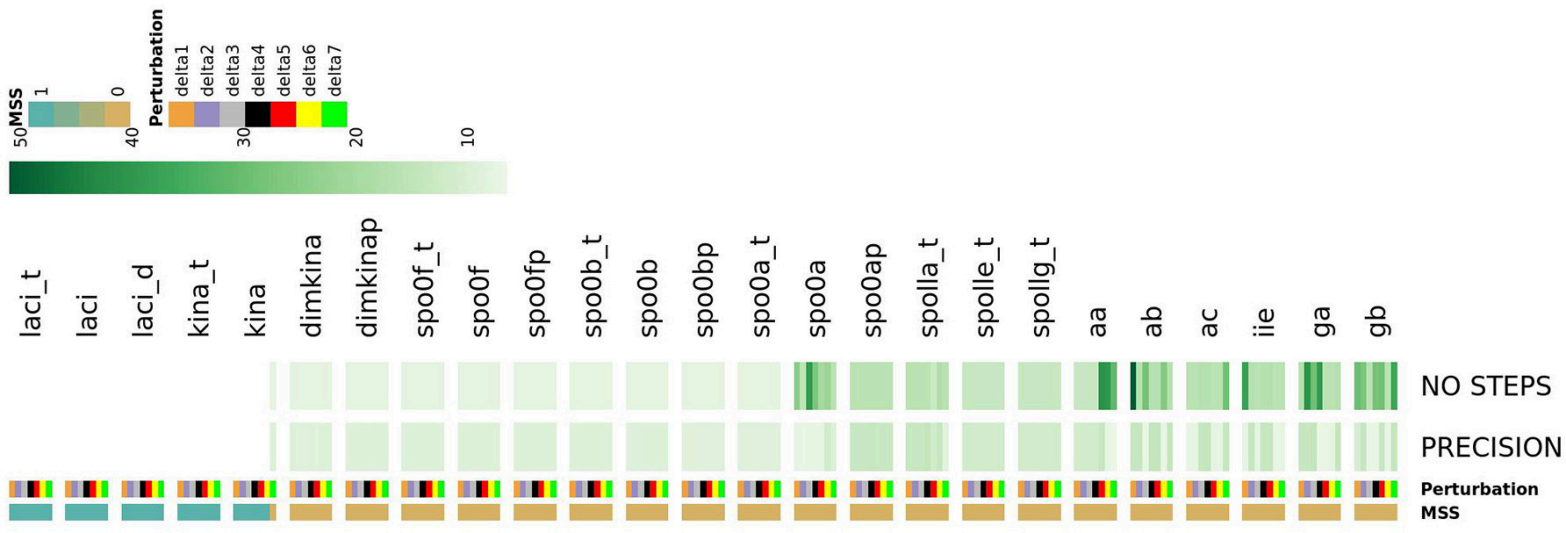
**The heatmap describes the systems ability to reach the steady state in response to seven different perturbation values of each species**. System's response is described by three features: MSS, PRECISION and NO STEPS. Depending on the perturbation magnitude of the initial state, the Newton-Raphson methods warns about the non-convergence to a steady state. This occurs when, depending on the initial conditions, the system has multiple steady states (MSS), and the Newton-Raphson solution methods flips unpredictability among them (Bloomfield, 2014). MSS is a binary feature assuming 1 if the system reaches multiple steady states and 0 otherwise; PRECISION is the precision in computation of a single steady state (reported in negative log scale.); NO STEPs is the number of steps required converge to a single steady state. Perturbations extent is shown for each systems species by a color bar. The map shows that the perturbation of laci_t, laci, laci_d, and kina causes an unresolved multi-stationarity.

As shown in Figure 14, this analysis allowed detecting a number of species which, once perturbed, can cause different types of complex evolutionary trajectories from the initial state of the system. Perturbing the initial state of the *laci_t, laci, laci_d, kina_t* and *kina* species caused the most noticeable consequence, preventing the system from reaching the steady state, irrespectively of the applied perturbation extent. This finding reflects the role of the species as starters of sporulation dynamics. For another subset of species, such as spo0a, aa, ab, ga, and gb, perturbing the initial state caused an increase in the number of iterative steps required by the Newton-Raphson method to reach the steady state, compared to the majority of the species in the system. This effect is indicative of increased complexity in the evolving system.

**Figure 15:**
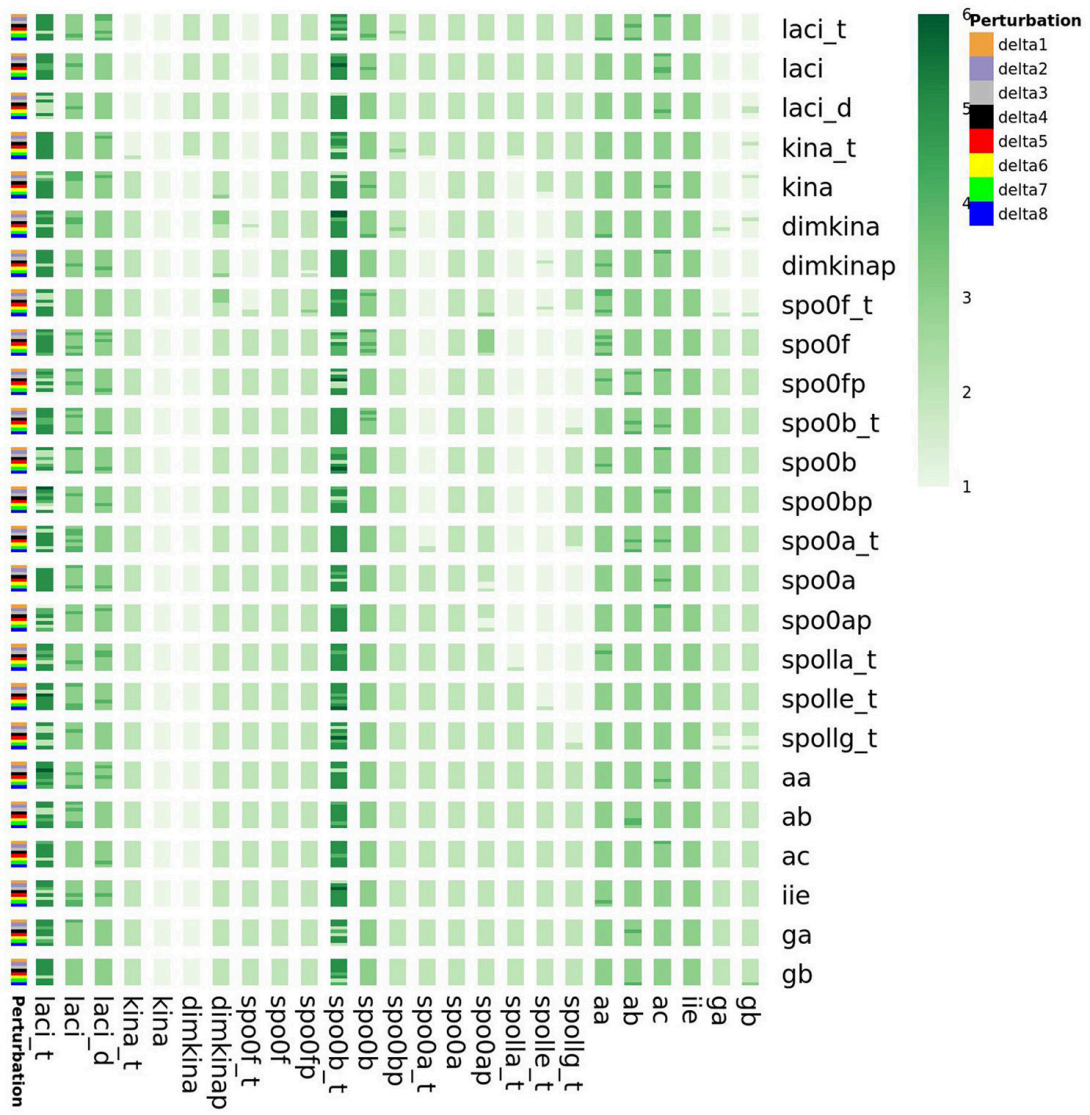
**Embedding dimension in response to perturbation of the initial state of the system variables**. On the vertical right axis, the name of perturbed species is reported, whereas on the left vertical axis the order of magnitude of the perturbation is reported. laci_t, laci, laci_d, kina_t, spo0b and spo0b_t embedding dimension is highly sensitive to perturbation of the initial conditions.

Furthermore, as shown in the Supplementary Material, we also found that the perturbations of the initial state of all the species cause variations in the minimum embedding dimension of spo0b, and spo0b_t, as well as of laci_t, laci, laci_d, kina_t (Figure 15). Quantification of the increase in minimum embedding dimension upon perturbation of the initial state is a further indication of the high sensitivity to perturbations, for the species highlighted by the initial analysis of complexity indices, such as spo0b, spo0b_t, and for the species emerging from the previous characterization of perturbation experiments.

Perturbations of initial conditions also cause variations of the Kaplan-Yorke ratio (i.e., $\left(\overset{j}{\underset{i=1}{\sum }}\lambda_{i}\right)/\lambda_{i+j}$, where *j* is the largest integer such that $\overset{j}{\underset{i=1}{\sum }}\lambda_{i}>0$) of spo0b_t, kina_t, and gb and of the Kolmogorov-Sinai entropy (i.e., the sum of positive Lyapunov exponents) of kina_t (Figures S8–S10). Hence, by multiple analyses the system showed a highly unpredictable behavior to perturbation of the initial state, as reflected by exponentially growing separation of the trajectories as well as by the topological complexity of the time series.

## Conclusions

We have presented a detailed, and original analysis of the *B. subtilis* sporulation initiation network dynamics. This analysis aimed to detect the presence of low-dimensional chaos in the dynamics of the system. Unlike more common approaches to chaos detection, this analysis includes and, is based on, a mathematical model of the *B. subtilis* sporulation initiation dynamics. This approach allows a comprehensive explanation of the mechanisms through which those molecular species with chaotic dynamics interact with the others and propagate the effects of chaos throughout the system.

Our analysis has: (i) assessed sensitivity of the dynamics by varying the kinetic parameters and the initial state of the model to determine the dynamic control parameters and identify the most crucial molecular species; (ii) calculated the complexity indices of the time series obtained from the model, and used these to identify the drivers of chaotic dynamics, and finally, (iii) calculated the Jacobian matrix of the system of equations as a function of time to find the steady state points and their nature to give an estimate of the number of active degrees of freedom of system as function of time.

We found that the dynamics of the *B. subtilis* sporulation initiation network is affected by low-dimensional chaos and identified Spo0B as the principal driver of the chaotic dynamics in this system. Spo0B scored positive for the majority of the chaos indices. This result suggests a new role for this molecular species, which so far has received little attention, and highlights the importance of its dynamics and interactions within the network model structure. Our analysis also indicates additional experimental work that could be conducted to improve our understanding of the sporulation network and to determine the role of Spo0B. On one hand, it would be important to conduct phosphoproteomics experiments to measure the amount of phosphorylated species, for which no experimental data is yet available. On the other hand, the study of Spo0B mutated (overexpressed/silenced) strains would refine our knowledge about the dynamics of the 3-level *B. subtilis* phosphorelay and the so far elusive mechanisms that could be regulating its expression. Other molecular species that also showed positive results for the test of chaos were *spo0a, spo0b_t, laci*, and *laci_t*. These molecular species also represent the degrees of freedom that are active during most of the range of the simulated time. These species have been identified as the drivers of the chaotic dynamics of *B. subtilis* sporulation initiation network model, and have an active role in determining its predictability.

One of the most challenging goal of studying a complex biological system is to control it. In this vein, our work proposes a method to identify the drivers in our *B. subtilis* sporulation initiation network on which such methods of chaos control might be applied. At the same time, our work highlights the need to investigate on these drivers and their mechanism of interaction in in order to successfully implement chaos control.

## Author contributions

All authors have equally contributed to the design and implementation of the mathematical analytical procedure, the discussion of the results and the writing of this paper.

## Funding

This work was partially supported by the Biotechnology and Biological Sciences Research Council (BBSRC) Institute Strategic Programme [BB/J004529/1]: The Gut Health and Food Safety ISP.

### Conflict of interest statement

The authors declare that the research was conducted in the absence of any commercial or financial relationships that could be construed as a potential conflict of interest.
