# Supplementary material for: Time Series Analysis of the Bacillus subtilis Sporulation Network Reveals Low Dimensional Chaotic Dynamics
Source: Front Microbiol. 2016 Nov 7;7:1760. doi: 10.3389/fmicb.2016.01760 (PMC5097912; doi:10.3389/fmicb.2016.01760)
Supplement: Supplementary file 1 [file Presentation1.pdf]

Supplementary Material: Time series analysis of the Bacillus subtilis sporulation network reveals low dimensional chaotic dynamics

Paola Lecca*, Ivan Mura, Angela Re, Gary Barker and Adaoha Ihekwaba*
*Correspondence:
Paola Lecca
Department of Mathematics, University of Trento
via Somamrive 14, 38123 Povo (Trento), Italy
paola.lecca@unitn.it

Adaoha Ihekwaba
Gut Health and Food Safety Institute of Food Research,
Norwich Research Park, Colney, Norwich NR4 7UA, UK
Adaoha.Ihekwaba@ifr.ac.uk

1 ORDINARY DIFFERENTIAL EQUATIONS AND PARAMETERS OF THE NETWORK MODEL

We report here the ordinary differential equations (ODEs) and the parameters of the model of the *B. subtilis* sporulation initiation network published in Ihekwaba et al. (2014). All state variables are in normal text, all parameters in italic. Parameter names are formed by a sequence of tokens separated by underscore characters ("_"). They begin with letter k for reaction rates, or Kk for Hill function parameters, or n for Hill function exponents, followed by a token that indicates the biological process, followed by a token that indicates the species the process is active upon, and possibly followed by a token that identifies a regulatory species (when it exists), or a partner species in the reaction (e.g., phosphotransfers). The only exception to this notation is for the mRNA and the protein degradation rates, which are denoted by $degm$ and $degp$, respectively, and which do not change depending on the species. As the concentration of the IPTG and SS species does not change over time, no differential equations exist in the model for these two species. The Tables S1, S2, and S3 collect all the parameters, explain their their biological meaning and report their value. The ODEs are given in Table S4. The time is measured in seconds, and the abundance of the species in nM. The model has been simulated on a time interval from 0 to 15,000 seconds with a time resolution of 10 seconds. Numerical solutions are shown in Figure S1.

Table S1. Parameters of the input signal sub-model

Parameter Name	Meaning	Value
k_tr_laci	rate of translation of lacI	0.1
degm	rate of mRNA degradation	9.0058
k_trl_laci	rate of lacI translation	0.2
k_re_laci	rate of lacI reactivation	0.01
k_in_laci	rate of lacI inactivation	0.00012
degp	rate of protein degradation	0.0208
k_trbasal_kina	basal rate of KinA transcription	0.24
k_tr_kina_laci	rate of lacI induced transcription of KinA	50
Kk_tr_kina_laci	Hill parameter for the lacI induced transcription of KinA	50
k_tr_kina_spo0a	rate of Spo0A induced transcription of KinA	1.95
Kk_tr_kina_spo0a	Hill parameter for the Spo0A induced transcription of KinA	2,100
k_dim_kina	rate of KinA dimerization	0.001
k_undim_kina	rate of KinA dimer dissociation	0.25
k_trl_kina	rate of KinA translation	0.0659
k_ph_kina	rate of KinA dimer phosphorylation	0.001

Table S2. Parameters of the phosphorelay sub-model.

Parameter Name	Meaning	Value
k_pht_kina_spo0f	rate of KinA dimer to Spo0F phosphotransfer	0.001
k_trl_spo0f	rate of Spo0F translation	0.0723
k_deph_spo0f	rate of Spo0F dephosphorylation	0.05
k_pht_spo0f_spo0b	rate of Spo0F to Spo0B phosphotransfer	0.001
k_trl_spo0b	rate of Spo0B translation	0.1076
k_pht_spo0b_spo0a	rate of Spo0B to Spo0A phosphotransfer	0.02
k_trl_spo0a	rate of Spo0A translation	0.2143
k_deph_spo0a	rate of Spo0A dephosphorylation	0.05
k1_tr_spo0a_spo0a	rate of Spo0A repressed transcription of Spo0A	0.013888
Kk1_tr_spo0a_spo0a	Hill parameter for the Spo0A repressed transcription of Spo0A	100
k2_tr_spo0a_spo0a	rate of Spo0A induced transcription of Spo0A	0.13888
Kk2_tr_spo0a_spo0a	Hill parameter for the Spo0A induced transcription of Spo0A	150
k_tr_spo0b	rate of Spo0B transcription	0.2384
k_tr_spo0f_spo0a	rate of Spo0A induced transcription of Spo0F	0.1
Kk_tr_spo0f_spo0a	Hill parameter for the Spo0A induced transcription of Spo0F	50

Table S3. Parameters of the gene expression sub-model.

Parameter Name	Meaning	Value
k_trbasal_spolla	basal rate of SpollA transcription	0.0277
k_tr_spolla_spo0a	rate of Spo0A induced transcription of SpollA	0.4166
n_tr_spolla_spo0a	Hill coefficient for Spo0A induced transcription of SpollA	4
Kk_tr_spolla_spo0a	Hill parameter for Spo0A induced transcription of SpollA	140
k_trbasal_spolle	basal rate of SpollE transcription	0.0208
k_tr_spolle_spo0a	rate of Spo0A induced transcription of SpollE	140
n_tr_spolle_spo0a	Hill coefficient for Spo0A induced transcription of SpollE	4
Kk_tr_spolle_spo0a	Hill parameter for Spo0A induced transcription of SpollE	230
k_trbasal_spollg	basal rate of SpollG transcription	0.0222
k_tr_spollg_spo0a	rate of Spo0A induced transcription of SpollG	0.729
n_tr_spollg_spo0a	Hill coefficient for Spo0A induced transcription of SpollG	4
Kk_tr_spollg_spo0a	Hill parameter for Spo0A induced transcription of SpollG	1,700
k_trl_aa	rate of AA translation	0.125
k_trl_ab	rate of AB translation	0.0555
k_trl_ac	rate of AC translation	0.138
k_trl_iie	rate of IIE translation	0.138
k_trl_ga	rate of GA translation	0.034
k_trl_gb	rate of GB translation	0.0138

Table S4. Ordinary differential equations describing the dynamics of *B. Subtilis* sporulation initiation network.

$$
\begin{aligned}
\frac{d}{dt}\mathrm{laci\_t} &= k\_tr\_laci - degm \cdot \mathrm{laci\_t} \\
\frac{d}{dt}\mathrm{laci} &= k\_trl\_laci \cdot \mathrm{laci\_t} + k\_re\_laci \cdot \mathrm{laci\_d} - k\_in\_laci \cdot \mathrm{laci} \cdot \mathrm{IPTG} - degp \cdot \mathrm{laci} \\
\frac{d}{dt}\mathrm{laci\_d} &= -k\_re\_laci \cdot \mathrm{laci\_d} + k\_in\_laci \cdot \mathrm{laci} \cdot \mathrm{IPTG} - degp \cdot \mathrm{laci\_d} \\
\frac{d}{dt}\mathrm{kina\_t} &= \left(k\_trbasal\_kina + \frac{k\_tr\_kina\_laci \cdot Kk\_tr\_kina\_laci^2}{Kk\_tr\_kina\_laci^2 + laci^2}\right) \cdot \left(1 + \frac{k\_tr\_kina\_spo0a \cdot \mathrm{spo0ap}^2}{Kk\_tr\_kina\_spo0ap^2 + \mathrm{spo0ap}^2}\right) \\
&\quad -degm \cdot \mathrm{kina\_t} \\
\frac{d}{dt}\mathrm{kina} &= -degp \cdot \mathrm{kinA} - 2 \cdot k\_dim\_kina \cdot \mathrm{kina} \cdot \mathrm{kina} + 2 \cdot k\_undim\_kina \cdot \mathrm{dimkina} \\
&\quad +2 \cdot degp \cdot \mathrm{dimkina} + 2 \cdot degp \cdot \mathrm{dimkinap} + 2 \cdot k\_undim\_kina \cdot \mathrm{dimkinap} \\
&\quad +k\_trl\_kina \cdot \mathrm{kina\_t} \\
\frac{d}{dt}\mathrm{dimkina} &= k\_dim\_kina \cdot \mathrm{kina} \cdot \mathrm{kina} - k\_undim\_kina \cdot \mathrm{dimkina} - 2 \cdot degp \cdot \mathrm{dimkina} \\
&\quad -k\_ph\_kina \cdot \mathrm{dimkina} \cdot \mathrm{SS} + k\_pht\_kina\_spo0f \cdot \mathrm{dimkinap} \cdot \mathrm{spo0f} \\
\frac{d}{dt}\mathrm{dimkinap} &= k\_ph\_kina \cdot \mathrm{dimkina} \cdot \mathrm{SS} - k\_pht\_kina\_spo0f \cdot \mathrm{dimkinap} \cdot \mathrm{spo0f} \\
&\quad -2 \cdot degp \cdot \mathrm{dimkinap} - k\_undim\_kina \cdot \mathrm{dimkinap} \\
\frac{d}{dt}\mathrm{spo0f} &= k\_trl\_spo0f \cdot \mathrm{spo0f\_t} - degp \cdot \mathrm{spo0f} - k\_pht\_kina\_spo0f \cdot \mathrm{dimkinap} \cdot \mathrm{spo0f} \\
&\quad +k\_deph\_spo0f \cdot \mathrm{spo0fp} + k\_pht\_spo0f\_spo0b \cdot \mathrm{spo0fp} \cdot \mathrm{spo0b} \\
\frac{d}{dt}\mathrm{spo0fp} &= k\_pht\_kina\_spo0f \cdot \mathrm{dimkinap} \cdot \mathrm{spo0f} - k\_deph\_spo0f \cdot \mathrm{spo0fp} \\
&\quad -degp \cdot \mathrm{spo0fp} - k\_pht\_spo0f\_spo0b \cdot \mathrm{spo0fp} \cdot \mathrm{spo0b} \\
\frac{d}{dt}\mathrm{spo0b} &= k\_trl\_spo0b \cdot \mathrm{spo0b\_t} - degp \cdot \mathrm{spo0b} - k\_pht\_spo0f\_spo0b \cdot \mathrm{spo0fp} \cdot \mathrm{spo0b} \\
&\quad +k\_pht\_spo0b\_spo0a \cdot \mathrm{spo0bp} \cdot \mathrm{spo0a} \\
\frac{d}{dt}\mathrm{spo0bp} &= k\_pht\_spo0f\_spo0b \cdot \mathrm{spo0fp} \cdot \mathrm{spo0b} - k\_pht\_spo0b\_spo0a \cdot \mathrm{spo0bp} \cdot \mathrm{spo0a} \\
&\quad -degp \cdot \mathrm{spo0bp} \\
\frac{d}{dt}\mathrm{spo0a} &= k\_trl\_spo0a \cdot \mathrm{spo0a\_t} - degp \cdot \mathrm{spo0a} - k\_pht\_spo0b\_spo0a \cdot \mathrm{spo0bp} \cdot \mathrm{spo0a} \\
&\quad +k\_deph\_spo0a \cdot \mathrm{spo0ap} \\
\frac{d}{dt}\mathrm{spo0ap} &= -degp \cdot \mathrm{spo0ap} + k\_pht\_spo0b\_spo0a \cdot \mathrm{spo0bp} \cdot \mathrm{spo0a} - k\_deph\_spo0a \cdot \mathrm{spo0ap} \\
\frac{d}{dt}\mathrm{spolla\_t} &= k\_trbasal\_spolla + \frac{k\_tr\_spolla\_spo0a \cdot \mathrm{spo0ap}^{n\_tr\_spolla\_spo0a}}{\mathrm{spo0ap}^{n\_tr\_spolla\_spo0a} + Kk\_tr\_spolla\_spo0a^{n\_tr\_spolla\_spo0a}} \\
&\quad -degm \cdot \mathrm{spolla\_t} \\
\frac{d}{dt}\mathrm{spolle\_t} &= k\_trbasal\_spolle + \frac{k\_tr\_spolle\_spo0a \cdot \mathrm{spo0ap}^{n\_tr\_spolle\_spo0a}}{\mathrm{spo0ap}^{n\_tr\_spolle\_spo0a} + Kk\_tr\_spolle\_spo0a^{n\_tr\_spolle\_spo0a}} \\
&\quad -degm \cdot \mathrm{spolle\_t} \\
\frac{d}{dt}\mathrm{spollg\_t} &= k\_trbasal\_spollg + \frac{k\_tr\_spollg\_spo0a \cdot \mathrm{spo0ap}^{n\_tr\_spollg\_spo0a}}{\mathrm{spo0ap}^{n\_tr\_spollg\_spo0a} + Kk\_tr\_spollg\_spo0a^{n\_tr\_spollg\_spo0a}} \\
&\quad -degm \cdot \mathrm{spollg\_t} \\
\frac{d}{dt}\mathrm{aa} &= k\_trl\_aa\_t \cdot \mathrm{spolla\_t} - degp \cdot \mathrm{aa} \\
\frac{d}{dt}\mathrm{ab} &= k\_trl\_ab\_t \cdot \mathrm{spolla\_t} - degp \cdot \mathrm{ab} \\
\frac{d}{dt}\mathrm{ac} &= k\_trl\_ac\_t \cdot \mathrm{spolla\_t} - degp \cdot \mathrm{ac} \\
\frac{d}{dt}\mathrm{iie} &= k\_trl\_iie\_t \cdot \mathrm{spolle\_t} - degp \cdot \mathrm{iie} \\
\frac{d}{dt}\mathrm{ga} &= k\_trl\_ga\_t \cdot \mathrm{spollg\_t} - degp \cdot \mathrm{ga} \\
\frac{d}{dt}\mathrm{gb} &= k\_trl\_gb\_t \cdot \mathrm{spollg\_t} - degp \cdot \mathrm{gb} \\
\frac{d}{dt}\mathrm{spo0a\_t} &= \frac{k1\_tr\_spo0a\_spo0a \cdot Kk1\_tr\_spo0a\_spo0a}{Kk1\_tr\_spo0a\_spo0a + \mathrm{spo0ap}} + \frac{k2\_tr\_spo0a\_spo0a \cdot \mathrm{spo0ap}^2}{Kk2\_tr\_spo0a\_spo0a^2 + \mathrm{spo0ap}^2} - degm \cdot \mathrm{spo0a\_t} \\
\frac{d}{dt}\mathrm{spo0b\_t} &= k\_tr\_spo0b - degm \cdot \mathrm{spo0b\_t} \\
\frac{d}{dt}\mathrm{spo0f\_t} &= k\_trbasal\_spo0f + \frac{k\_tr\_spo0f\_spo0a \cdot \mathrm{spo0ap}^2}{Kk\_tr\_spo0f\_spo0a^2 + \mathrm{spo0ap}^2} - degm \cdot \mathrm{spo0f\_t}
\end{aligned}
$$

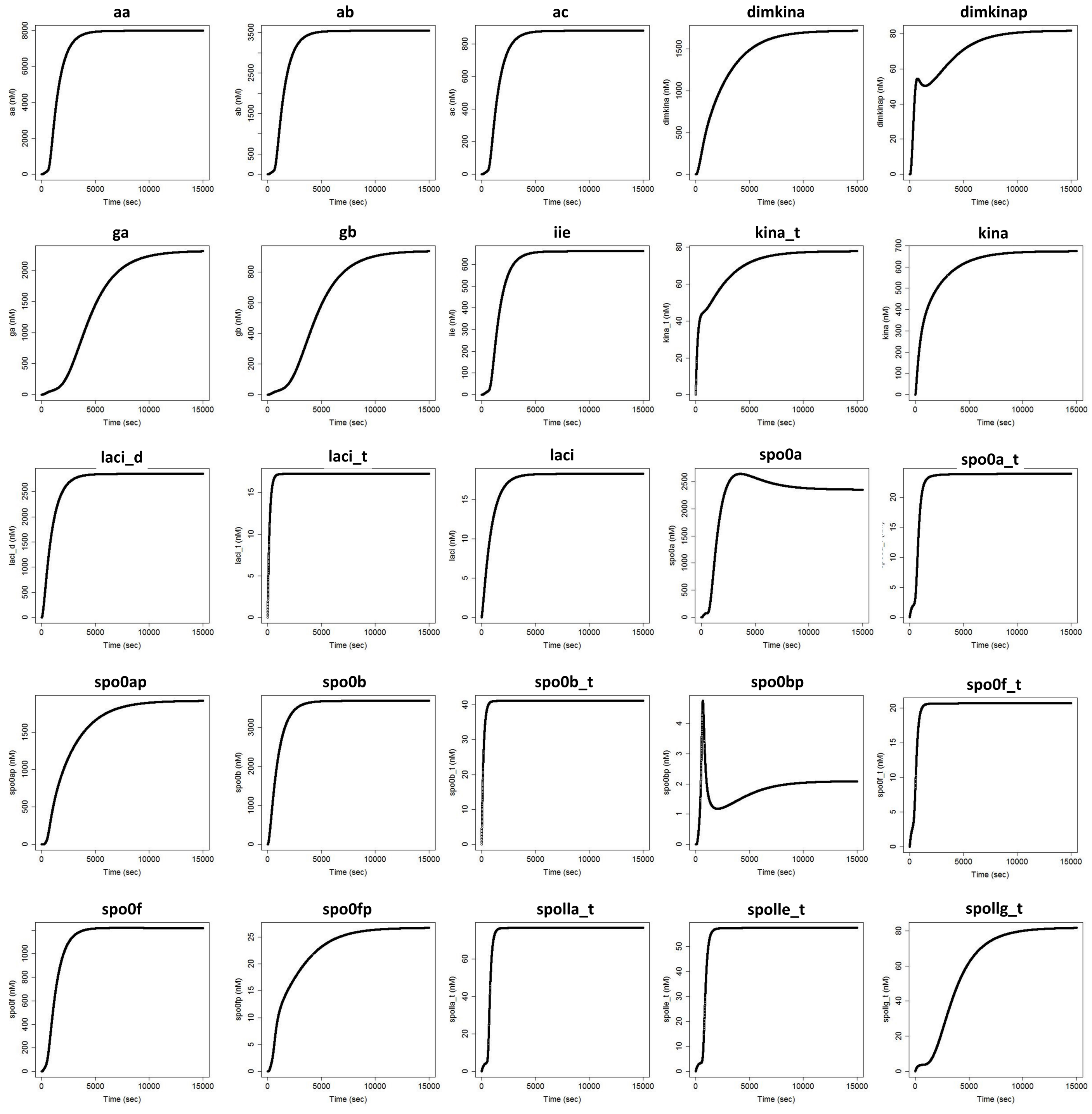

Figure S1. Numerical solutions of the ODEs system. Most molecular species show a steep monotonic behaviour, which reflects the stiffness of system's dynamics.

2 SUPPLEMENTARY ANALYSES

This section collects the results of (i) the sensitivity analysis performed on a smaller range of parameter variation (Figure S2); (ii) the comparison between complexity indices estimated from the time series and for those estimated from coloured and power-spectrum noise (Figures S4, S5, and S6), that confirm that the complex behavior is due to chaos rather than to noise, and (iii) the recurrence plots (Figure S7). Table S5 reports the results of the the recurrence quantification analysis (RQA). RQA quantifies the number and duration of recurrences of the state space trajectories of a dynamical system (Marwan et al., 2016).

2.1 Sensitivity analysis

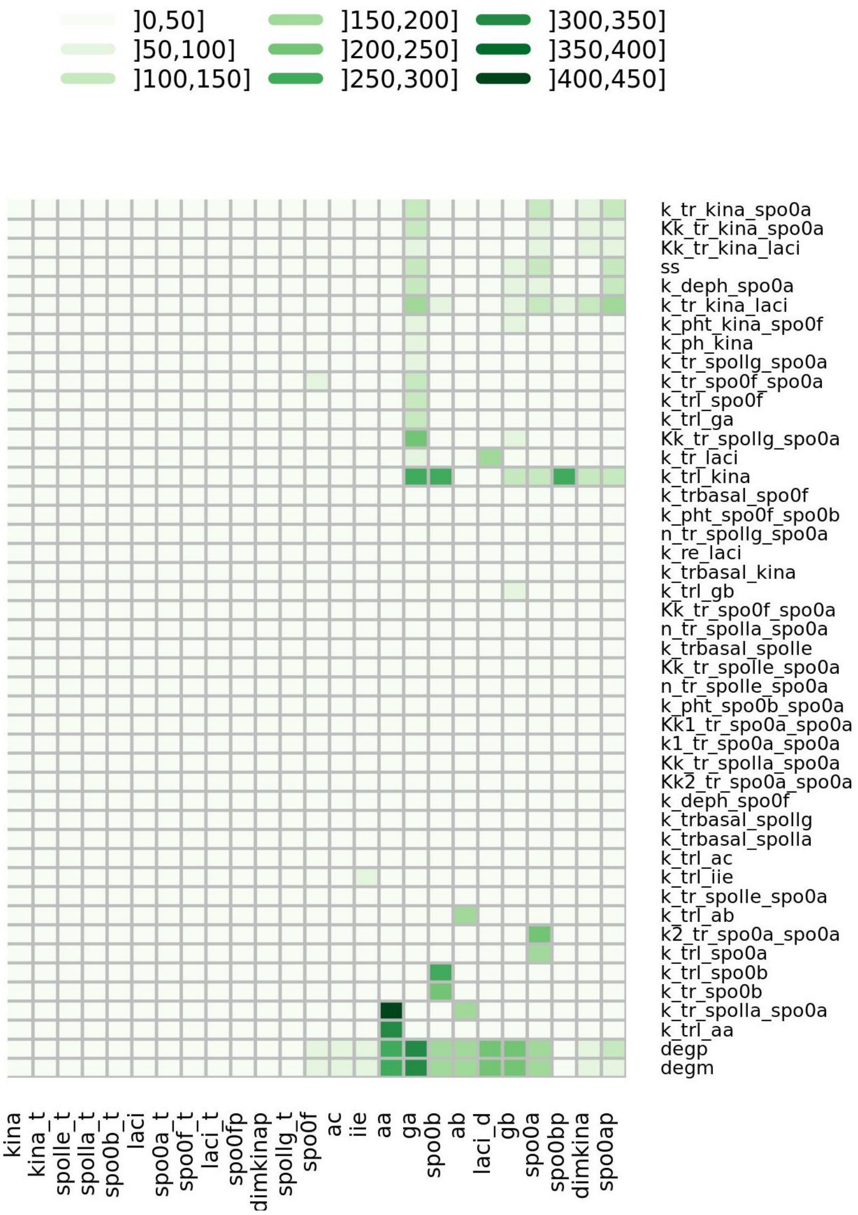

Figure S2. Heatmap summarizing the results of sensitivity analysis. The size of the interval of parameter variation is defined by $q = 2$ in Eq. 1. The most sensitive molecular species are $aa, ga, spo0b, ab, laci\_d, gb, spo0a$. They are sensitive to the 50% of the parameters.

2.2 Analysis of monotonicity

The (non-decreasing) monotonicity is the property for which $\frac{\partial s_i}{\partial t} \geq 0, \forall i | \mathbf{\Theta} \in \mathbb{R}^{N_P}$, where s_i is the solution of the equation for the i-th molecular species, and $\mathbf{\Theta}$ is the set of parameter values.

In an interval of parameter variation defined as in Eq. 13, we samples 10,000 parameter configurations, and for each solution s_i we calculated the percentage of time points at which $\frac{\partial s_i}{\partial t} \geq 0$. Formally, the Proportion of Positive Derivatives values (PPD) for the species i is.

$$PPD_i = \frac{1}{10,000} \sum_{i=1}^{10,000} \left(\sum_{k=1}^{N} \frac{PD_i(t_k)}{N} \right)$$

where, N is the length of the time series (here it is equal for all the species), and

$$PD_i = \begin{cases} 1 & \text{if } \left.\frac{\partial s_i}{\partial t}\right|_{t=t_k} \geq 0 \\ 0 & \text{otherwise} \end{cases}$$

The derivative has been calculated with the Stineman method (Johannesson and Bjornsson, 2012). The results are shown in Figure S3 A. For 20 species out of 25 the derivative is invariably positive. The negative time derivatives of the solutions are due to (i) small fluctuations/irregularities of the curves (for *spo0b* and *spo0f*); (ii) a slow slight decreasing of the curve before reaching a plateau (for *spo0a*); (iii) a rapid increment/decrement on a short time interval and/or of small magnitude (for *dimkinap*, and *spo0bp*). The plots in Figures S3 B, C, D, E, and F show that the standard deviation of the simulation curves is approximately null and that their overall trend is monotone.

2.3 Steep monotonicity and global sensitivity analysis

A steep monotonic behaviour reflects the stiffness of the system's equations, which is due to the presence of substantially different orders of magnitude of the parameters (see Table S3). The stiffness of the system warns about the accuracy of the estimates of Sobol indices (Sumner et al., 2012) that quantify the global parameter sensitivity of the model. It is known that numerical methods for integrating stiff equations on large integration domain tend to be inaccurate. Since Sobol indices computation requires to integrate the solutions of the equations in the parameters space (Sumner et al., 2012), the occurrence of stiff equations would imply a severe loss of accuracy of the Sobol indices for our systems. The integrals in the definition of Sobol indices are usually evaluated using Monte Carlo integration methods that use random or quasi-random sampling of the model parameters. To obtain reliable accurate estimates of the Sobol indices, especially for stiff systems, the sample size should be taken large (i.e. the step size of the integration should be taken extremely small). However, the complexity of the method scales as $N_S(N_P + 2)$, where N_S is the sample size and N_P is the number of parameters. As reported in (Morio, 2011) ten thousands samples are often necessary for the estimation of one Sobol index with a relative error of 10%. In order to have smaller relative errors, we should increase N_S. However, if the system is highly stiff in the parameters space, as it is in our case, N_S should be extremely high, so that the use of Sobol method becomes impractical (Sumner et al., 2012). Therefore, we set out to preferentially avoid global sensitivity analysis in our study.

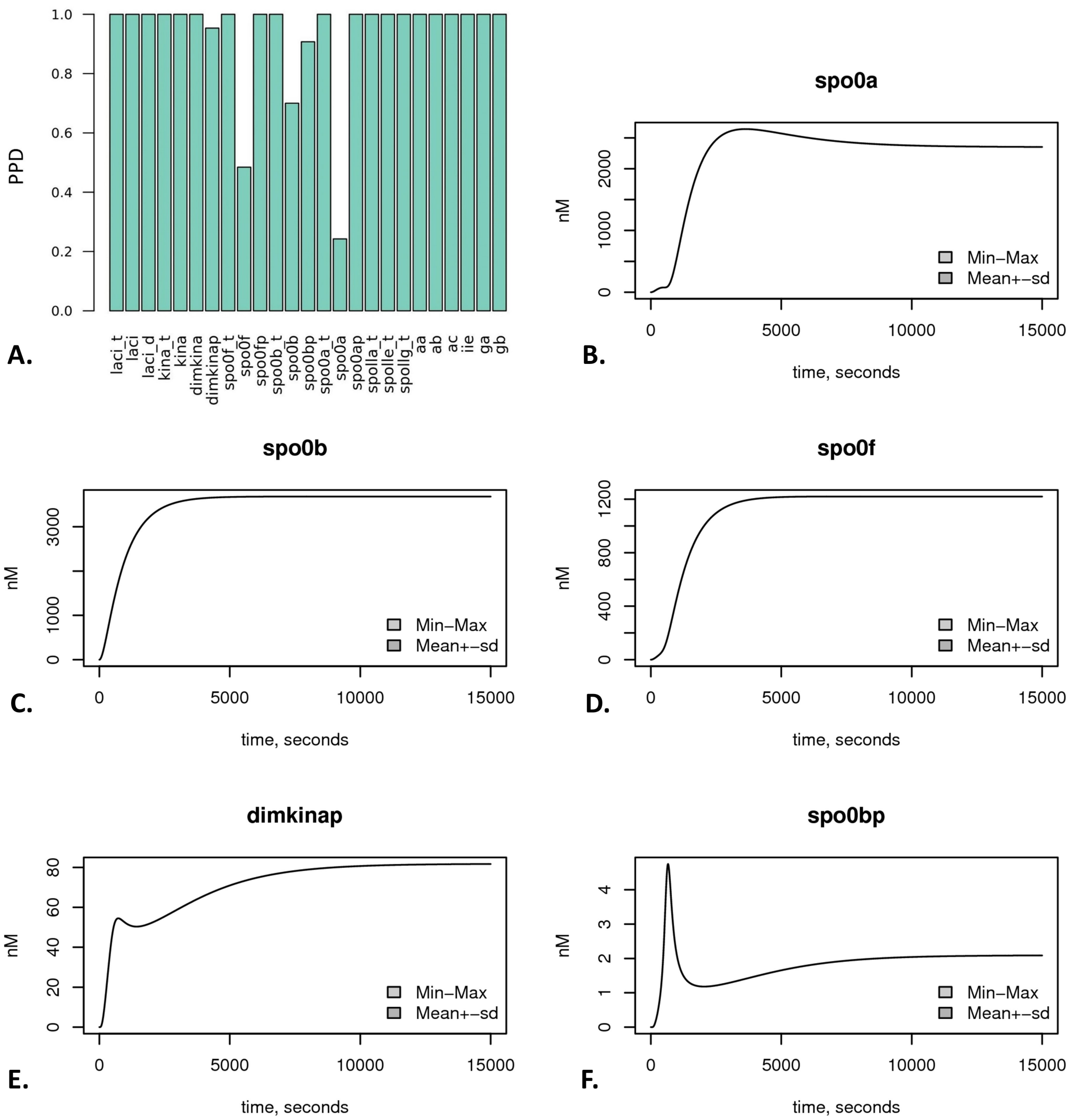

Figure S3. Analysis of monotonicity. **A.** Proportion of time points at which the time derivative of the numerical solution of the dynamical of a species is positive or null. **B - F** Simulation curves for the species whose time derivative assumes also negative values. All the parameters have been changed over the range defined in Eq. (13), and the effect on these variables has been assessed. The values of the parameters have been sampled from a uniform distribution positively defined on this range. The model has been ran 5,000 times. An upper (max) and a lower (min) bound simulation curve, along with the mean simulation curve and its standard deviation have been calculated (with the literature algorithms presented in (Soetaert et al., 2016a,b, 2010)). The plots show that these curves overlap, as the standard deviation is very close to zero. Therefore, these simulation prove that the model is monotonic and that it does not respond to a global sensitivity analysis approach to parameter perturbation. Finally, note that although the behavior of *spo0bp* shows vlear deviations from monotonicity, the magnitude of the *spo0bp* variation amounts to only few nMs. Indeed, *spo0b* rapidly increases from 0 to only 4 nM and then it decays to 1 nM from which it starts to monotonically increase, whereas the range on y-axis covered by the other species in the same simulation time is tens/thousand times larger.

2.4 Distinguishing chaos from noise

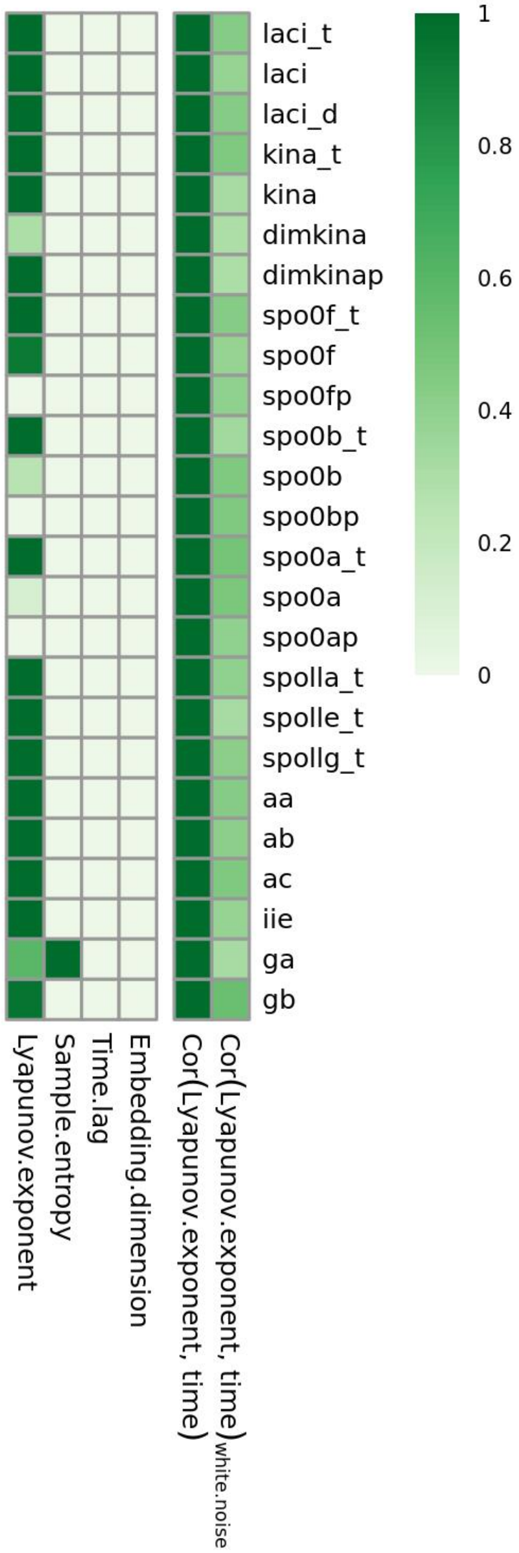

Figure S4. Complexity indices (right-handside heatmap) and linear correlation coefficient significance for the time behaviour of Lyapunov exponents (left-handside heatmap) estimated for the model time series and for a pink noise signal.

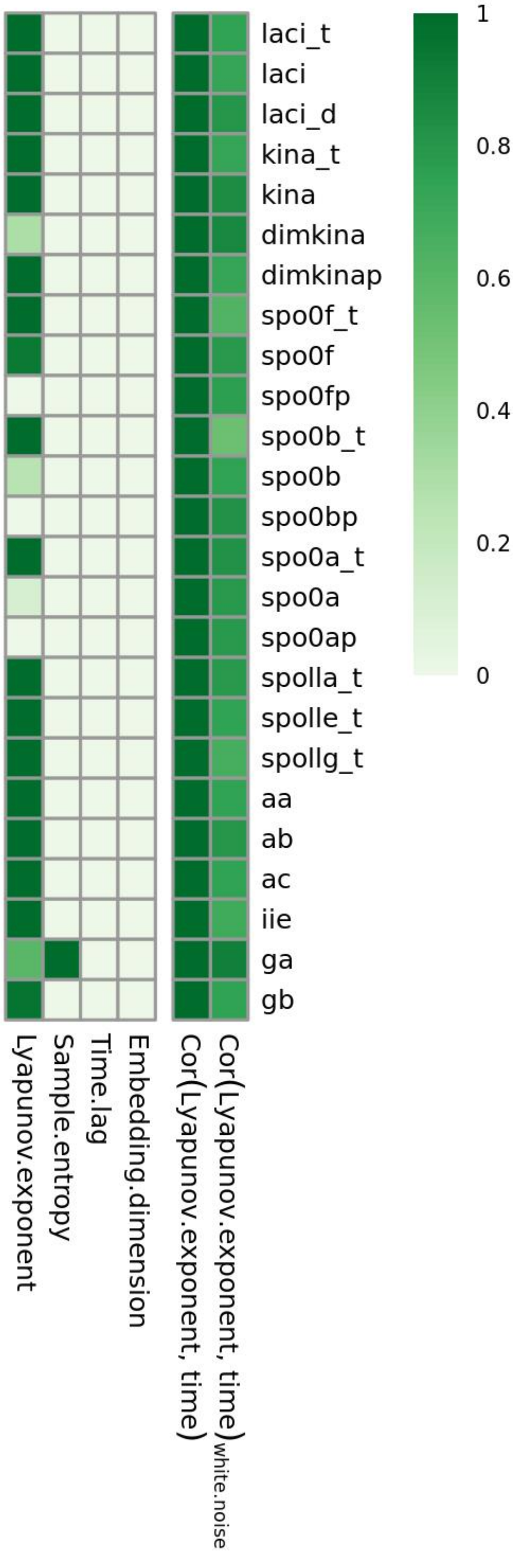

Figure S5. Complexity indices (right-handside heatmap) and linear correlation coefficient significance for the time behaviour of Lyapunov exponents (left-handside heatmap) estimated for the model time series and for a red noise signal.

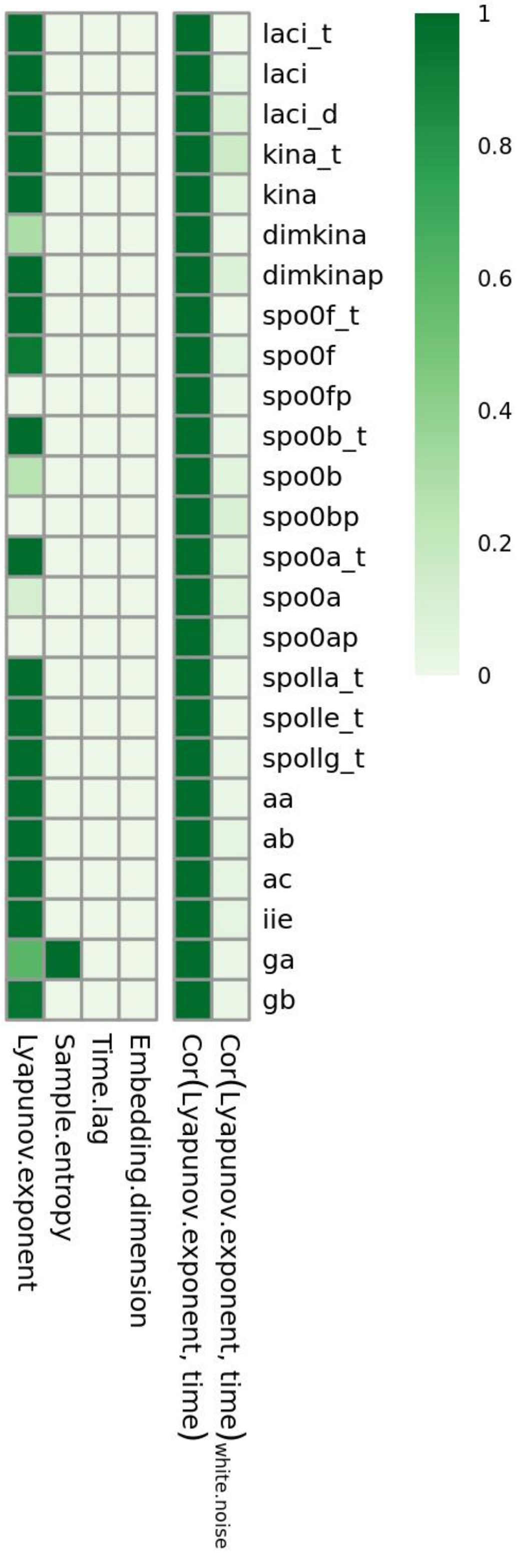

Figure S6. Complexity indices (right-handside heatmap) and linear correlation coefficient significance for the time behaviour of Lyapunov exponents (left-handside heatmap) estimated for the model time series and for a power-low spectrum noise signal.

2.5 Recurrence plots (RPs)

A d-dimensional phase space trajectory ($d > 2$) can be visualized through a two-dimensional representation of its recurrences.

The recurrence plot (RP), proposed by Eckerman et al. (Eckmann et al., 1987), employs a two-dimensional squared matrix of zero values, where both axes are time axes. The recurrence of a state, observed at time i, at a time j different from i is marked with one (black dot in the plot) in the matrix. In formulas, an RP can be expressed as follows:

$$\mathbf{R}_{i,j} = \Theta(\nu_i - ||\mathbf{x}_i - \mathbf{x}_j||), \quad \mathbf{x}_i \in \mathbb{R}^d, \quad i, i = 1, \ldots, N$$

where N is the length of the time series $\mathbf{x}$, ν_i is a threshold distance, $|| \cdot ||$ is a norm, and $\Theta(\cdot)$ is the Heaviside step function

$$\Theta(z) \begin{cases} 0 & x < 0 \\ \frac{1}{2} & z = 0 \\ 1 & z > 0 \end{cases}$$

The RPs structures are indicative of the time evolution of phase space trajectories. A comprehensive introduction to RPs and the interpretation of their structure is given in (Marwan et al., 2016; Abraham et al., 1989). Here, we summarize the main definitions and concepts as reported in (Marwan et al., 2016, 2002).

The RPs structure is characterized by large scale (typologies) and small scale patterns (textures).

The typologies in a RP can be (i) a homogeneous, (ii) a periodic, and (iii) a disrupted distribution of recurrent points.(Eckmann et al., 1987). Homogeneous RPs are common to chaotic and to stochasitc dynamics. Diagonally oriented, periodic recurrent structures (diagonal lines, checkerboard structures), represent oscillating systems. Fading to the upper left and lower right corners indicate non-stationarity (i.e. the presence of a drift or trend). Finally, white areas or bands in RP are typical in presence of abrupt changes in the dynamics as well as of rare events.

The textures can be single dots, diagonal lines as well as vertical and horizontal lines.

- Single, isolated recurrent points can occur if states are rare, if they do not persist for any time or if they fluctuate heavily.
- A diagonal line $\mathbf{R}_{i+k,j+k} = 1$ (for $k = 1, \ldots, l$, where l is the length of the diagonal line) occurs when a segment of the trajectory runs parallel to another segment, i.e. the trajectory visits the same region of the phase space at different times. The length of this diagonal line is determined by the duration of such similar local evolution of the trajectory segments. In presence of diagonal lines, the process coudl be deterministic with no chaos; if these diagonal lines occur beside single isolated points, the process could be affected by deterministic chaos (if these diagonal lines are periodic, unstable periodic orbits can be retrieved).
- A vertical (horizontal) line $\mathbf{R}_{i,j+k} = 1$ (for $k = 1, \ldots, v$, where v is the length of the vertical line) marks a time length in which a state does not change or changes very slowly. It seems, that the state is trapped for some time. This is a typical behaviour of laminar states (intermittency).

The textures of a RP are the base of the definition of measure for a quantitative analysis of the RPs, called *Recurrence Quantification Analysis* (RQA) (Webber et al., 2016; Marwan et al., 2002; Trulla et al., 1996).

The list of the RQA measures, a short explanation of their meaning, and their values we found for the variables of the *B. subtilis* sporulation initiation network, is given in Table S5. DET measures the proportion of recurrent points forming diagonal line structures parallel to the main diagonal. Lmax, i.e. the length of the longest diagonal line, inversely scales with the maximal Lyapunov exponent (Eckmann et al., 1987; Trulla et al., 1996). Positive Lyapunov exponents gauge the rate at which trajectories diverge, and are the hallmark for dynamic chaos. Thus, the shorter the Lmax, the more chaotic (less stable) the signal. ENTR is the Shannon entropy of the distribution of the length of line segments parallel to the main diagonal. ENTR is a measure of signal complexity and is calibrated in units of bits/bin to quantify how much information one needs in order to recover the system. The entropy is small when the length of the longest segment parallel to the diagonal is short and does not vary much. This has to be associated with information on determinism. A high entropy is typical of periodic behavior while low entropy indicates chaotic behavior (Fabretti and Ausloos, 2004; Blackledge et al., 2002).

Table S5 shows that DET is equal to 100% for all the molecular species, that means that for all the molecular species all the recurrent points lie on diagonal segments parallel to the main diagonal. This is a typical characteristic of deterministic systems (with and without chaos). However, we also found a significant variability in the mean length (Lmean) of these segments, and more than 50% of the molecular species with a Lmean smaller than the means value. These molecular species are marked in bold in Table S6. The same set of species exhibits values of Vmean and ENTR below the mean. Of the species indicated in bold in the table, spo0b, spo0bp, spo0a, spo0ap, aa, and ab, also report a value of Lmax below that the mean. There results confirm again spo0b as a species affected with chaotic dynamics.

Table S5. Summary of the recurrence quantification analysis (RQA). RQA quantifies the number and duration of recurrences of a dynamical system presented by its state space trajectory (Zbilut and Webber, 2006). The complexity indices estimated in RQA are as follows. **REC:** recurrence. Percentage of recurrence points in a Recurrence Plot. **DET:** Determinism. Percentage of recurrence points that form diagonal lines. **LAM:** Laminar states. Percentage of recurrent points that form vertical lines. **RATIO:** Ratio between DET and REC. **Lmax:** Length of the longest diagonal line. **Lmean:** Mean length of the diagonal lines. The main diagonal is not taken into account. **Vmax:** Longest vertical line. **Vmean:** Average length of the vertical lines. This parameter is also referred to as the Trapping time. **ENTR:** Shannon entropy of the diagonal line lengths distribution **TREND:** Trend of the number of recurrent points depending on the distance to the main diagonal. TREND measures how the density of points changes as you move away from the diagonal.

Variable	REC	DET	LAM	RATIO	Lmax	Lmean	Vmax	Vmean	ENTR	TREND
laci_t	0.9367	1	1	1.0675	7100	3437.9311	6930	6651.7022	8.8349	-1e-04
laci	0.69	1	1	1.4493	6700	2806.1381	5805	4623.5748	8.6148	-1e-04
laci_d	0.2357	1	0.9999	4.2428	5477	1578.8137	3253	1708.0701	7.9942	-1e-04
kina_t	0.2512	1	1	3.9802	7500	2120.9898	3996	1884.6019	8.0883	-1e-04
**kina**	0.0722	1	1	13.8473	7385	1462.127	1969	548.1873	7.233	0
**dimkina**	0.0151	1	0.9997	66.2752	6695	1158.0381	578	111.1783	5.7956	0
**dimkinap**	0.1655	1	1	6.0426	7100	1633.9865	2964	1154.1871	7.6307	-1e-04
spo0f_t	0.8701	1	1	1.1493	7500	3465.3561	7062	6526.4747	8.8484	-1e-04
spo0f	0.3719	1	1	2.6887	6428	2151.4713	4429	2855.3171	8.3412	-1e-04
spo0fp	0.3062	1	1	3.2664	7100	2226.0215	4173	2173.9841	8.1337	-1e-04
spo0b_t	0.9061	1	1	1.1036	6300	3000.7364	6055	5709.5431	8.6987	-1e-04
**spo0b**	0.2562	1	0.9999	3.9025	5771	1731.0719	3587	1991.606	8.1068	-1e-04
**spo0bp**	0.917	1	1	1.0905	7100	1689.21	6706	3276.0912	7.0922	-1e-04
spo0a_t	0.809	1	1	1.2361	7100	3168.9958	6489	5744.8933	8.756	-1e-04
**spo0a**	0.0142	1	0.9984	70.3931	5969	1168.553	540	119.7977	5.7263	0
**spo0ap**	0.0135	1	0.9991	74.034	6459	1148.5582	527	104.7233	5.6885	0
spolla_t	0.8134	1	1	1.2294	7100	3182.0755	6466	5775.7743	8.7614	-1e-04
spolle_t	0.7918	1	1	1.263	7100	3142.3768	6394	5622.2925	8.7477	-1e-04
**spollg_t**	0.1202	1	1	8.3176	7100	1528.2029	2496	853.7365	7.5264	0
**aa**	0.1649	1	0.9998	6.0646	5141	1419.2515	2904	1439.6496	7.8667	-1e-04
**ab**	0.1791	1	0.9998	5.5831	5080	1392.4322	2847	1399.3177	7.8454	-1e-04
ac	0.2921	1	1	3.424	6102	1852.0501	3831	2060.3351	8.1713	-1e-04
iie	0.2963	1	1	3.3752	6700	1868.8112	3860	1985.3856	8.1775	-1e-04
**ga**	0.006	1	1	165.2942	7500	948.1588	277	45.3794	5.1612	0
**gb**	0.0111	1	1	90.3412	7100	1071.3033	433	78.6017	5.5533	0

Table S6. Summary of the distributions of the typologies showed in Table S5.

	REC	DET	LAM	RATIO	Lmax	Lmean	Vmax	Vmean	ENTR	TREND
Min.	0.0060	1	0.9984	1.067	5080	948.2	277	45.38	5.161	-1.0e-04
1st Qu.	0.1202	1	0.9999	1.263	6300	1419.3	2496	853.74	7.233	-1.0e-04
Median	0.2562	1	1.0000	3.902	7100	1731.1	3831	1985.39	8.088	-1.0e-04
Mean	0.3802	1	0.9999	21.626	6664	2014.1	3783	2577.78	7.656	-7.2e-05
3rd Qu.	0.7918	1	1.0000	8.318	7100	2806.1	6055	4623.57	8.615	0.0e+00
Max.	0.9367	1	1.0000	165.294	7500	3465.4	7062	6651.70	8.848	0.0e+00

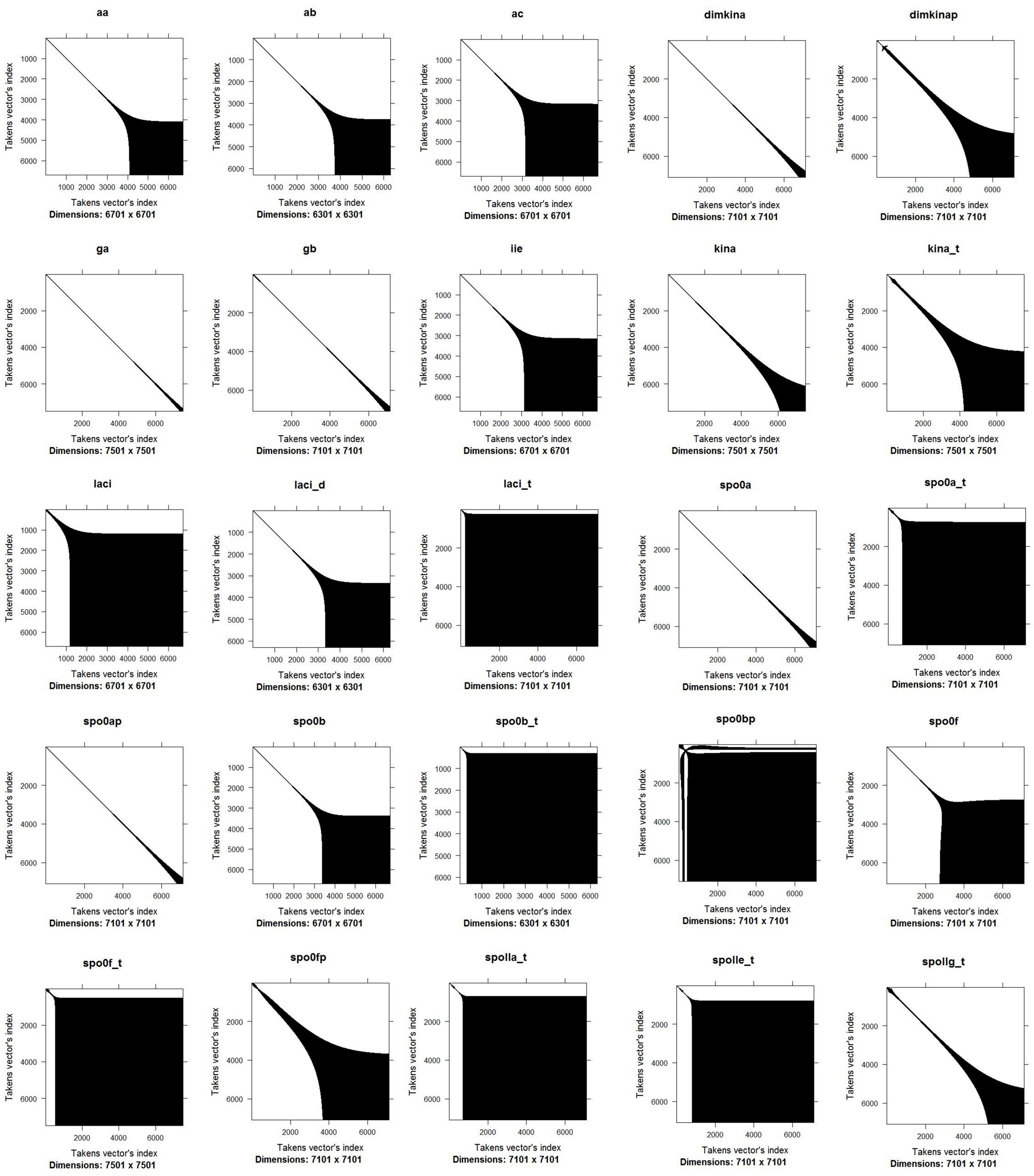

Figure S7. Global aspect of the RPs for the variables of the *B. subtilis* sporulation initiation network.

2.6 Kaplan-Yorke ratio and Kolmogorov-Sinai entropy

By virtue of their definition including the Lyapunov exponents, Kaplan-Yorke ratio (i.e. the second term of the Kaplan-Yorke dimension (Nichols et al., 2004; Hilborn, 2000)) and Kolmogorov-Sinai entropy (Hilborn, 2000), also known as metric entropy, are the indices expressing topological complexity as function of sensitivity of the systems to the initial conditions. Kaplan-Yorke ratio is defined as:

$$KY_R = \frac{\sum_{i=1}^{j} \lambda_i}{\lambda_{i+j}} \tag{S1}$$

where λ_i is the i-th Lyapunov exponent, and, ordering in decreasing order the Lyapunov exponents $\lambda_1 > \lambda_2 > \cdots > \lambda_i > \cdots > \lambda_d$, j is the largest integer such that $\sum_{i=1}^{j} \lambda_i > 0$.

Kolmogorov-Sinai entropy is defined as

$$KS_E = \sum_{\lambda_i > 0} \lambda_i. \tag{S2}$$

We calculated the values of KY_R and KS_E corresponding to the values of perturbations $\Delta = \{10^{-5}, 10^{-4}, 10^{-3}, 10^{-2}, 10^{-1}, 1, 10\}$ applied to one variable at a time. Figure S8 shows the boxplots of the distributions of the values KY_R and KS_E. The name of perturbed variable is indicated on the horizontal axis. The heatmaps in Figures S9 (S10) report respectively, the value of the difference between the mean values of KY_R (KS_E) distributions and the p-values of the multiple t-test performed to asses the statistical significance of this difference.

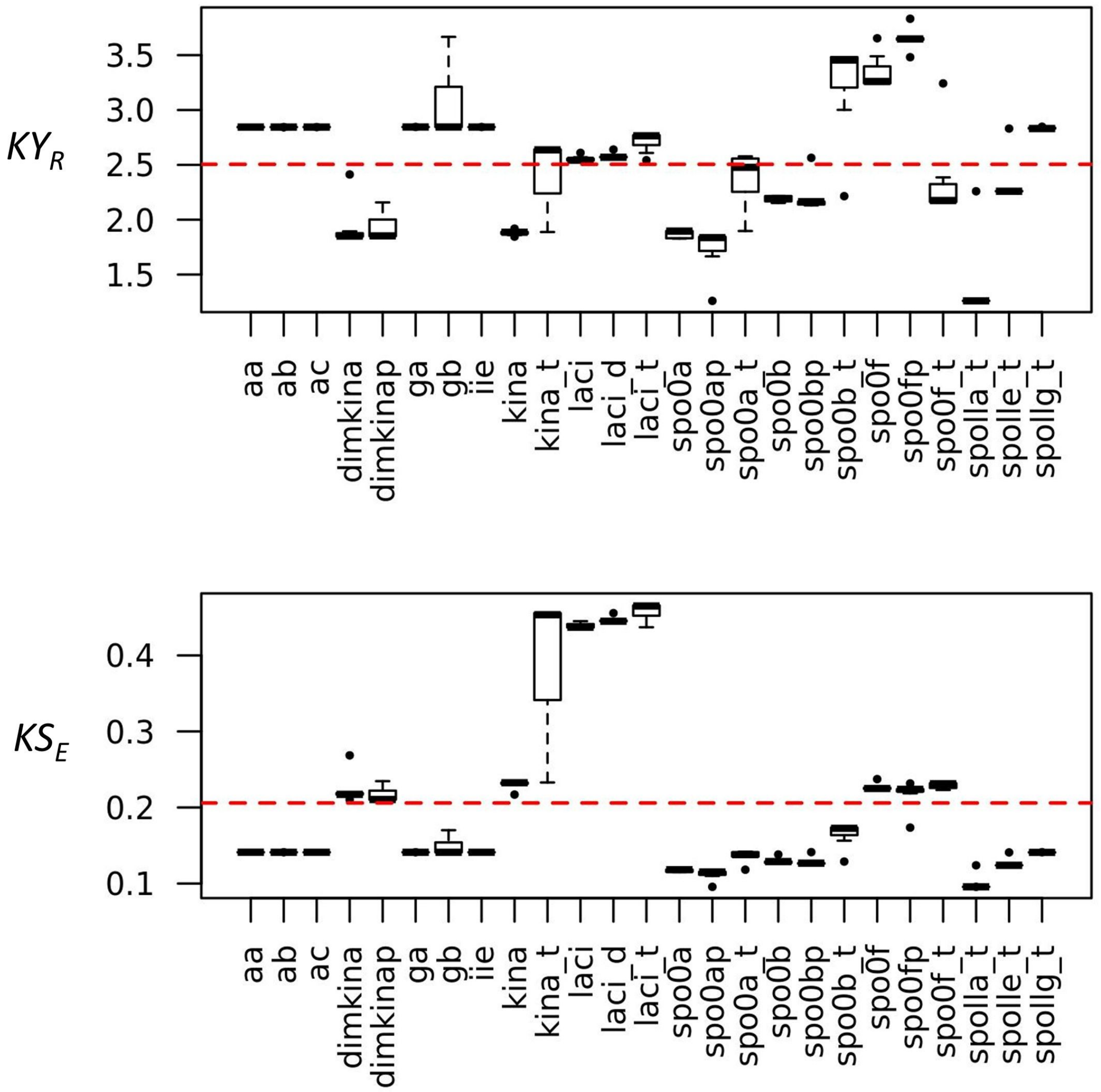

Figure S8. Boxplot showing the Kaplan-Yorke ratio and Kolmogorov-Sinai entropy corresponding to the perturbations of each species in the system. The dashed red line indicates the average values overall perturbations.

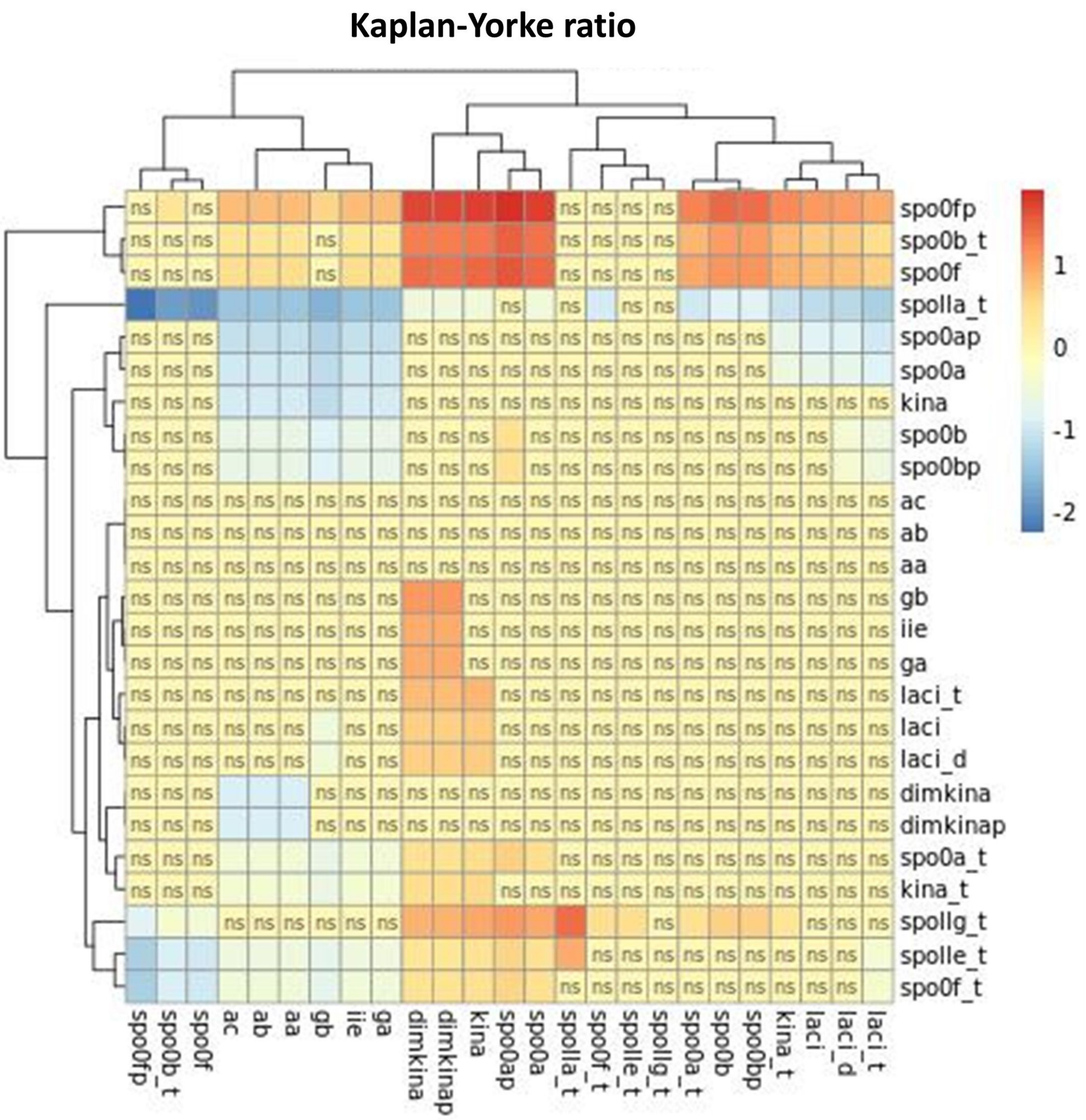

Figure S9. Heatmap showing the difference of means of the Kaplan-Yorke dimension corresponding to the perturbation of each pair of species in the system. Shown differences are computed by the Tukeys Honest Significant Difference method. P-values are shown after adjustment for the multiple comparisons. Not significant adjusted p-values are indicated in the heat maps cells by the abbreviation 'ns'.

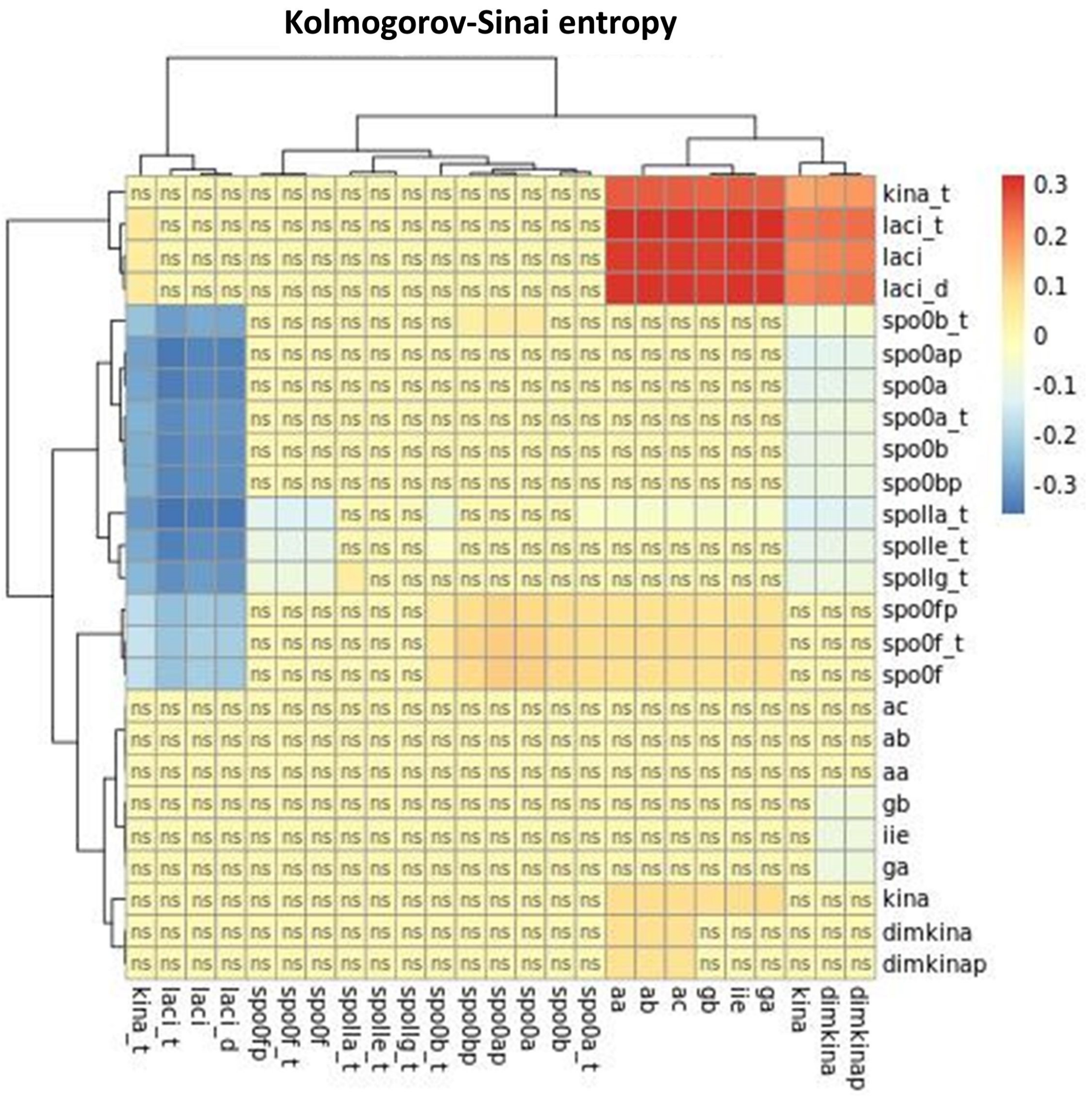

Figure S10. Heat map showing the difference of means of the Kolmogorov-Sinai entropy corresponding to the perturbation of each pair of species in the system. Shown differences are computed by the Tukeys Honest Significant Difference method. P-values are shown after adjustment for the multiple comparisons. Not significant adjusted p-values are indicated in the heat maps cells by the abbreviation 'ns'.

3 SOFTWARE

All the computational modules of the analysis presented in this paper have been implemented in R language (https://www.r-project.org/) and is available upon request. The real and the CPU times (in seconds) to process 7,500 time points and 25 time series are: 59754.43, and 36.78, on a Windows 8.1 Notebook PC, Intel Core i7, 16Gb RAM, and 3.1 GHz, and 17237.85, and 28.19 on a Windows 8.1 Desktop PC, Intel Core i3, 16Gb RAM, and 3.6 GHz. The most computationally expensive modules are those for the estimation of recurrence plots, sample entropy, and fractal dimension.

REFERENCES

Abraham, N. B., Albano, A. M., Passamante, A., and Rapp, P. E. (eds.) (1989). *Measures of Complexity and Chaos* (New York, NY, USA: Springer)

Blackledge, J. M., Evans, A., and Turner, M. J. (eds.) (2002). *Fractal Geometry: Mathematical Methods, Algorithms, Application* (Woodhead Publishing)

Eckmann, J. P., Kamphorst, S. O., and Ruelle, D. (1987). Recurrence plots of dynamical systems. *Europhys. Lett.* 4, 973977

Fabretti, A. and Ausloos, M. (2004). Recurrence plot and recurrence quantification anslysis techniques for detecting critical regime. Arxiv: https://arxiv.org/ftp/cond-mat/papers/0412/0412765.pdf

Hilborn, R. (2000). *Chaos and Nonlinear Dynamics: An Introduction for Scientists and Engineers* (Oxford: Oxford University Press)

Ihekwaba, A., Mura, I., and Barker, G. C. (2014). Computational modelling and analysis of the molecular network regulating sporulation initiation in Bacillus subtilis. *BMC Systems Biology* 8, 119. doi:10.1186/s12918-014-0119-x

Johannesson, T. and Bjornsson, H. (2012). Stineman, a consistently well behaved method of interpolation. http://rpackages.ianhowson.com/cran/stinepack/

Marwan, N., Romano, M., and Thiel, M. (2016). Recurrence plots and cross recurrence plots. Http://www.recurrence-plot.tk/

Marwan, N., Wessel, N., Meyerfeldt, U., Schirdewan, A., and Kurths, J. (2002). Recurrence-plot-based measures of complexity and their application to heart-rate-variability data. *Phys. Rev. E.* 66, 026702

Morio, J. (2011). Global and local sensitivity analysis methods for physical systems. *Eur. J. Phys.* 32, 15771583

Nichols, J. M., Todd, M. D., Seaver, M., Trickey, S. T., Pecora, L. M., and Moniz, L. (2004). Controlling system dimension: A class of real systems that obey the kaplanyorke conjecture. *Proc Natl Acad Sci USA* 100, 1529915303

Soetaert, K., Petzoldt, T., and Setzer, R. W. (2010). Inverse Modelling, Sensitivity and Monte Carlo Analysis in R Using Package FME. *Journal of Statistical Software* 33, 1–28

Soetaert, K., Petzoldt, T., and Setzer, R. W. (2016a). deSolve: General Solvers for Initial Value Problems of Ordinary Differential Equations (ODE), Partial Differential Equations (PDE), Differential Algebraic Equations (DAE), and Delay Differential Equations (DDE). R package: URL http://CRAN.R-project.org/package=deSolve

Soetaert, K., Petzoldt, T., and Setzer, R. W. (2016b). R Package FME : Inverse Modelling, Sensitivity, Monte Carlo - Applied to a Dynamic Simulation Model. https://cran.r-project.org/web/packages/FME/vignettes/FMEdyna.pdf

Sumner, T., Shepard, E., and Bogle, I. D. L. (2012). A methodology for global-sensitiviy analysis of time-dependent outputs in systems biology modelling. *J. R. Soc. Interface* 9, 21562166

Trulla, L. L., Giuliani, A., Zbilut, J. P., and Webber, C. L. (1996). Recurrence quantification analysis of the logistic equation with transients. *Phys. Lett. A* 223, 255260

Webber, C. L., Ioana, C., and Marwan, N. (eds.) (2016). *Recurrence Plots and Their Quantifications: Expanding Horizons. Proceedings of the 6th International Symposium on Recurrence Plots, Grenoble, France, 17-19 June 2015* (Switzerland: Springer International Publishing)

Zbilut, J. P. and Webber, C. L. (2006). *Recurrence quantification analysis.* (Wiley Encyclopedia of Biomedical Engineering)
