# Supplementary figures and images for: Time Series Analysis of the Bacillus subtilis Sporulation Network Reveals Low Dimensional Chaotic Dynamics
Source: Front Microbiol. 2016 Nov 7;7:1760. doi: 10.3389/fmicb.2016.01760 (PMC5097912; doi:10.3389/fmicb.2016.01760)

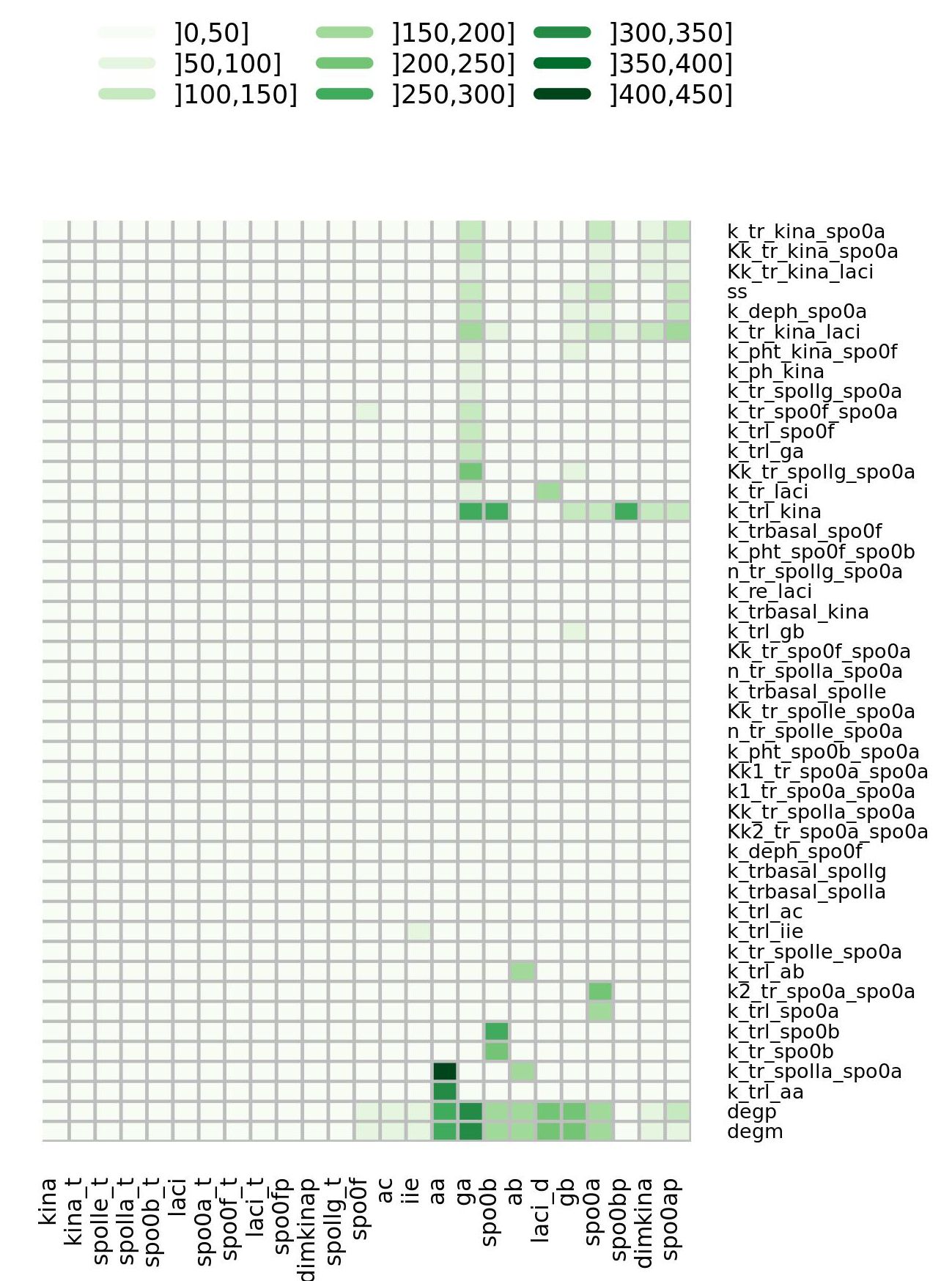

Supplement: Supplementary file 2 [file Presentation2.ZIP › Latex_Supplementary/Figure_S1.jpg]

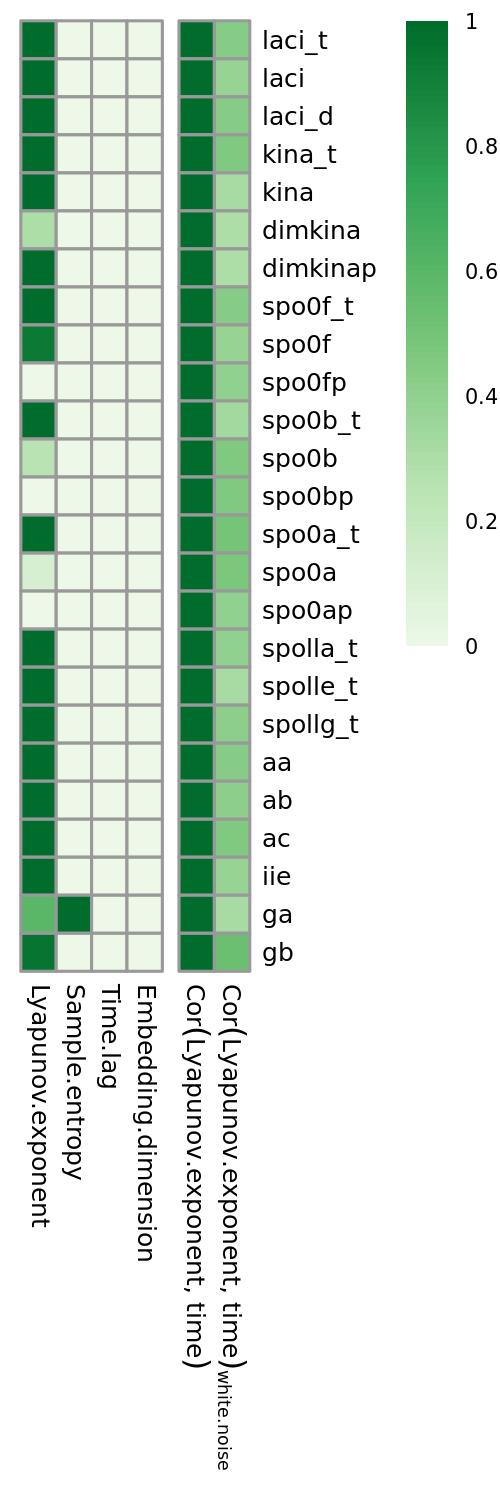

Supplement: Supplementary file 2 [file Presentation2.ZIP › Latex_Supplementary/Figure_S2.jpeg]

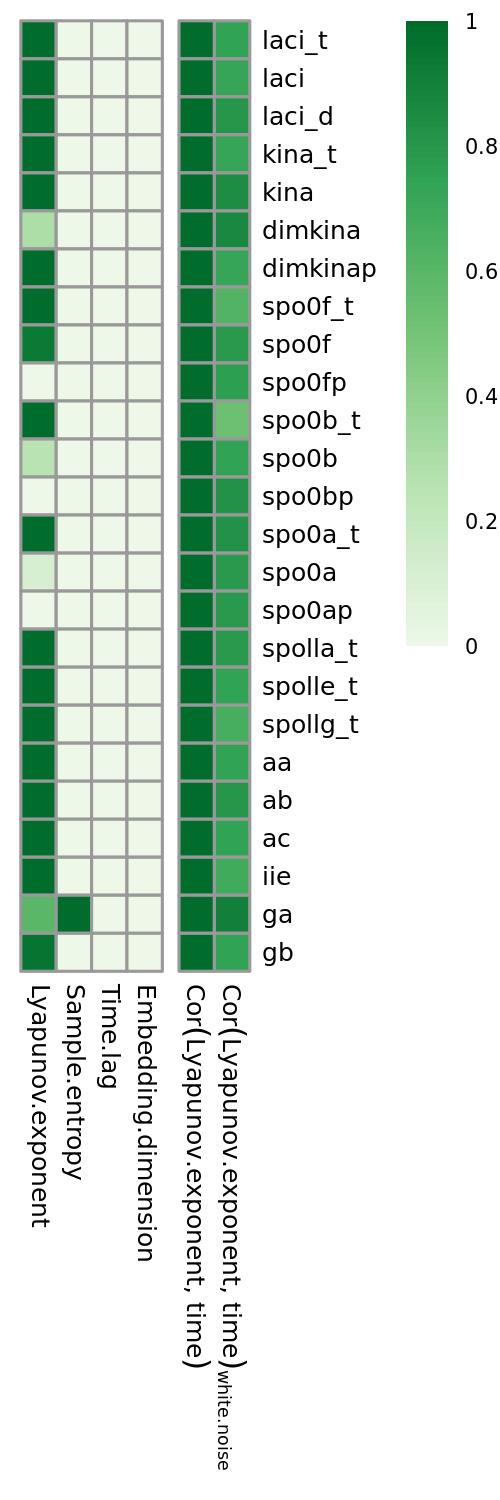

Supplement: Supplementary file 2 [file Presentation2.ZIP › Latex_Supplementary/Figure_S3.jpeg]

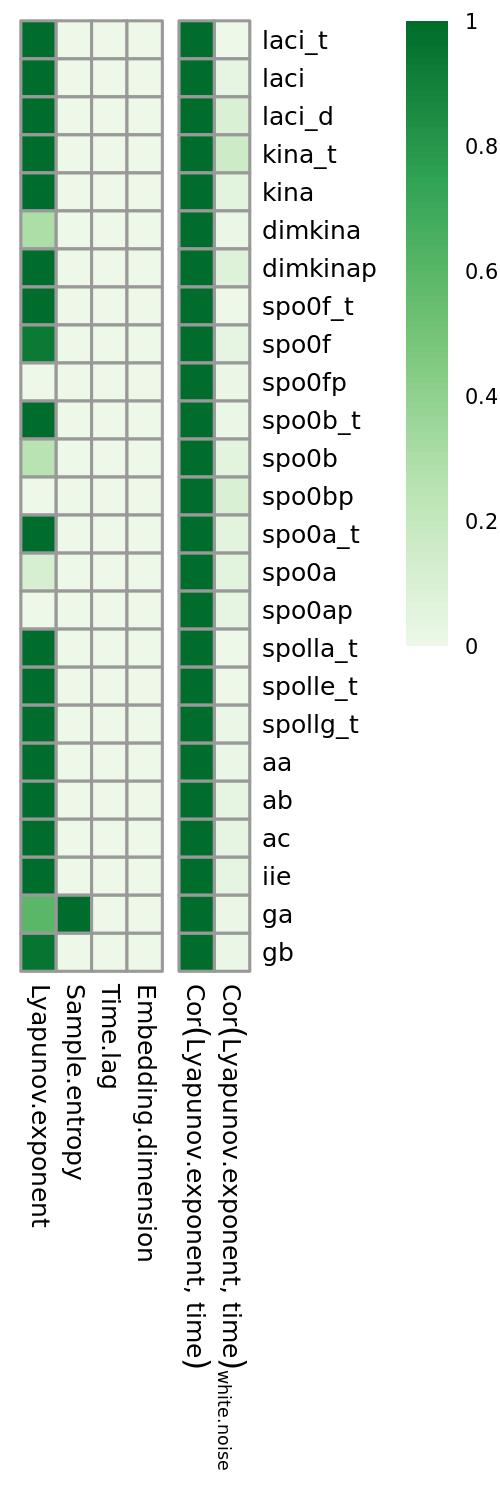

Supplement: Supplementary file 2 [file Presentation2.ZIP › Latex_Supplementary/Figure_S4.jpeg]

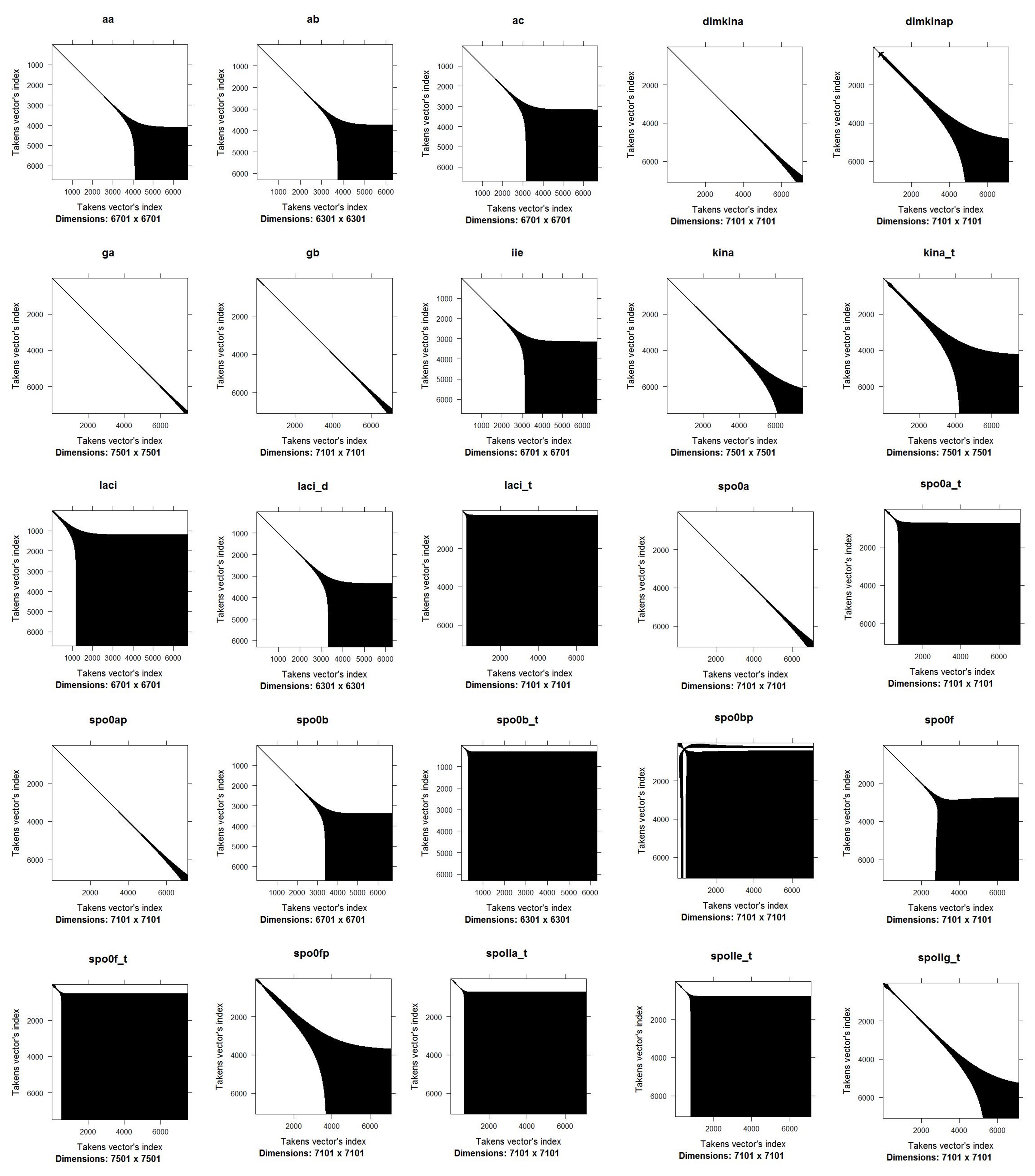

Supplement: Supplementary file 2 [file Presentation2.ZIP › Latex_Supplementary/Figure_S5.jpg]
